# Exosomes: a promising microenvironment modulator for spinal cord injury treatment

**DOI:** 10.7150/ijbs.115242

**Published:** 2025-06-05

**Authors:** Yanming Ma, Xiaojun Yu, Jingxin Pan, Yingguang Wang, Ruoyu Li, Xiaodong Wang, Huimin Hu, Dingjun Hao

**Affiliations:** Department of Spine Surgery, Honghui Hospital, Xi'an Jiaotong University, Xi'an, Youyidong Road, Shaanxi, 710054, China.

**Keywords:** Spinal Cord Injury, Exosomes, Microenvironment Modulator, Mechanism

## Abstract

Spinal cord injury (SCI) remains a severely disabling disorder that impacts millions globally by causing irreversible damage to the nervous system. Although cell - based therapies have shown notable progress, the post - injury microenvironment presents significant obstacles that hinder the survival and effectiveness of implanted cells, ultimately limiting sustained functional restoration. Exosomes have emerged as a promising cell - free therapeutic alternative due to their stability, low immunogenicity, and ability to carry bioactive molecules such as proteins, microRNAs, and lipids. These vesicles can modulate the injured microenvironment, support neuroprotection, and facilitate repair. This review begins by discussing the pathological alterations that disrupt the microenvironment following SCI. The review then outlines the process of exosome formation and highlights their structural features. Furthermore, the review delves into the diverse cellular sources of exosomes and evaluates their therapeutic relevance in the context of SCI. Special attention is given to the multifaceted roles exosomes play in neuroprotection, such as reinforcing the blood - spinal cord barrier, stimulating axonal regeneration, promoting new blood vessel formation, suppressing programmed cell death in neurons, and modulating inflammatory responses. The synergistic use of exosomes in combination with biomaterials is also explored, with the aim of optimizing their therapeutic potential. Lastly, the review addresses the key obstacles that must be overcome to bring exosome - based treatments into clinical application and offers perspectives on future advancements in this evolving field. In summary, exosomes offer a novel and promising avenue for SCI intervention, holding considerable promise as an alternative to traditional therapeutic approaches.

## Introduction

Spinal cord injury (SCI) is a catastrophic condition of the central nervous system, ranking among the foremost causes of long - term disability and mortality. It involves damage to the spinal cord that leads to the loss of sensory, motor, or autonomic function 1. SCI can cause partial or complete deficits below the site of injury, resulting in varying levels of impairment 2. According to estimates by the World Health Organization, between 250,000 and 500,000 new SCI cases occur globally each year 3. Data from the Global Burden of Disease Study in 2019 reported that approximately 20.6 million individuals were living with SCI. Notably, around 90% of these injuries are caused by external trauma, including traffic collisions, falls, and violent encounters 4. Initial mechanical insults, also named as primary injury, result in immediate neuronal damage, vascular disruption, and breakdown of the blood - spinal cord barrier (BSCB), which includes contusion, compression, and severance. This is rapidly followed by secondary injury processes, which amplify the damage through glial activation, neuronal and oligodendrocyte apoptosis, and further inflammatory responses. Since primary injury is irreversible, understanding and mitigating the cascade of secondary injury remains the cornerstone of SCI treatment. Nevertheless, there remains no clinically validated method capable of reversing the chronic neurological deficits caused by SCI. The intricacy of post - injury pathophysiology creates a highly detrimental environment that significantly impairs axonal regeneration and limits recovery of function 5. Consequently, advancing our understanding of SCI mechanisms and developing innovative treatment modalities remains a pressing need.

Over the past decade, cell transplantation has emerged as a promising avenue to facilitate neural repair in SCI. Both neural and mesenchymal cell types have been explored, with mounting evidence indicating their potential to preserve axons, stimulate remyelination, and enhance motor recovery by modifying the pathological microenvironment 6-10. Despite substantial progress in transplantation techniques, the unfavorable milieu within the damaged spinal cord continues to challenge the survival and integration of grafted cells 11. To improve outcomes, researchers have investigated the mechanisms by which transplanted cells confer therapeutic benefits. For example, mesenchymal stem cells (MSCs) secrete a range of bioactive molecules, including cytokines and exosomes, that help regulate inflammation and support tissue repair 12-14. Olfactory ensheathing cells (OECs) are also under investigation due to their capacity to promote axonal regrowth, reduce neuropathic pain, and interact with astrocytes through immunomodulatory and neurotrophic mechanisms. 15, 16.

Mounting evidence indicates that the regenerative effects of transplanted cells may be largely attributed to the exosomes they release. Exosomes are nanoscale vesicles produced by a variety of cells and serve as essential mediators of intercellular signaling 17, 18. Typically ranging from 30 to 150 nanometers in size, these vesicles are formed within multivesicular endosomes and released into the extracellular space 19. Enclosed by a lipid bilayer, exosomes safeguard their cargo from enzymatic degradation, which may include proteins, lipids, and nucleic acids. This structural advantage allows them to influence target cells and modulate biological processes effectively 20. Owing to their inherent biocompatibility, stability, and ability to traverse biological barriers, exosomes are gaining traction as potential therapeutic agents in SCI 21, 22. Their low immunogenicity makes them suitable for clinical applications, and they can be engineered to deliver therapeutic molecules with high specificity 23, 24. Consequently, exosomes represent a promising alternative to traditional cell therapy, offering a novel platform for drug delivery and targeted regenerative interventions 25.

In this review, we begin by summarizing the alterations in the spinal cord microenvironment after SCI. Subsequently, we elucidate the origin and biological functions of exosomes. Additionally, we provide a comprehensive overview of the therapeutic applications of exosomes in SCI, highlighting current limitations and discussing future directions for research and therapeutic advancement.

## The microenvironment imbalance of SCI

After SCI, a disrupted equilibrium emerges across tissue, cellular, and molecular domains of the microenvironment 26. At the tissue level, this dysregulation manifests through events such as hemorrhagic damage, reduced blood supply, degradation and partial restoration of myelin, and the development of fibrotic or glial scars 27. On the cellular scale, pathological responses include the infiltration of immune cells, activation of resident microglia, loss of neurons, and the proliferation of reactive astrocytes 28-30. At the molecular level, the imbalance is marked by altered expression profiles of neurotrophic factors and their precursors, along with shifts in cytokine and chemokine signaling 31, 32. Collectively, these changes demonstrate a shift toward the downregulation of protective factors and the upregulation of detrimental factors following SCI.

### Tissue imbalance

**Figure 1:**
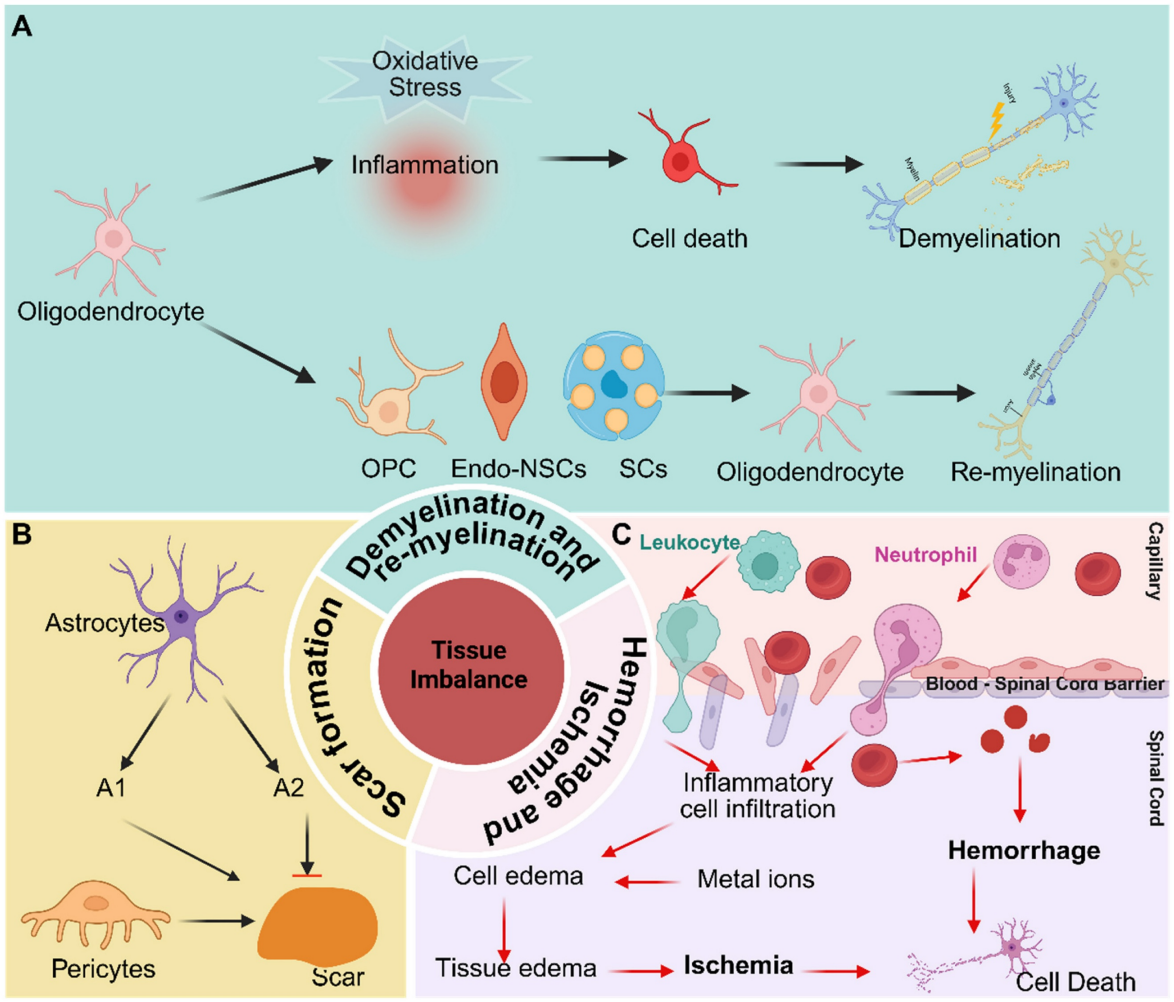
Tissue imbalance of microenvironment after SCI, including demyelination and re - myelination, hemorrhage and ischemia, and scar formation. Created with BioRender.com.

Tissue imbalance after SCI denotes the disruption of spinal cord homeostasis following injury, primarily involving hemorrhage and ischemia, demyelination and remyelination, and scar formation. (**Figure 1**).

#### Hemorrhage and Ischemia

BSCB provides a specialized microenvironment essential for maintaining the homeostasis of the spinal cord parenchyma. When the BSCB is compromised, this environment is disrupted, leading to hemorrhagic and ischemic imbalances 27. Local microvascular rupture directly exposes spinal cord tissue to blood, which contains various metal ions - particularly iron - that can induce neuronal ferroptosis 33. Venous stasis and distension can result in the buildup of protein - rich fluid within the tissue, leading to edema. Moreover, neural tissue edema elevates interstitial pressure, compressing adjacent blood vessels and further contributing to ischemia 34. In addition, rupture of supply vessels leads to cellular hypoxia, which in turn causes cellular edema, thereby exacerbating overall tissue swelling.

#### Demyelination and re - myelination

Myelin is essential for maintaining axonal integrity, which in turn facilitates the conduction of axonal signals. Following SCI, oligodendrocyte death results from both direct mechanical damage and an imbalance in the local microenvironment, ultimately leading to demyelination. Potential causes of oligodendrocyte death include mechanical injury, ischemia, pro - inflammatory cytokines, oxidative stress, and excitotoxicity mediated by glutamate and ATP 35, 36. Mounting evidence suggests that oligodendrocyte death during the acute phase of SCI is primarily mediated through apoptosis and necrosis. Apoptosis can occur as early as 6 hours after SCI in rats and may persist for up to 3 weeks in monkeys, contributing significantly to axonal demyelination 37. Necrosis, which typically occurs within the first 24 hours post - injury, triggers inflammatory responses 38. Additionally, several studies have shown that autophagy is induced in oligodendrocytes after SCI 39, 40. However, autophagy does not appear to be a significant contributor to oligodendrocyte loss in this context.

Conversely, remyelination naturally occurs after SCI, primarily through the replacement of oligodendrocytes. These newly formed oligodendrocytes originate from three principal sources: oligodendrocyte progenitor cells (OPCs), Schwann cells (SCs), and endogenous neural stem cells (Endo - NSCs). Studies have shown that OPCs in the injury core decrease within the first 24 hours post - injury; however, they are subsequently activated, rapidly proliferate, migrate, and differentiate into mature oligodendrocytes 26, 30, 41-43. Among them, OPCs are the primary source of oligodendrocytes in the injured spinal cord site. Nevertheless, myelin restoration is often incomplete following SCI. Microenvironmental disturbances which includes myelin debris, activation of the MBP signaling pathway, and the presence of pro - inflammatory factors significantly inhibit the differentiation of OPCs and the maturation of new oligodendrocytes. Numerous studies have reported the presence of SCs after SCI. Some indicate that these SCs are primarily derived from nerve roots, with only a small fraction (< 10%) originating from peripheral nerves. Approximately 70% - 80% of SCs, however, arise from resident OPCs, regardless of recombination efficiency 43. Despite this, the precise functions and mechanisms of newly generated SCs remain to be fully elucidated. In adult mammals, Endo - NSCs are primarily located in the subventricular zone, the hippocampal subgranular zone, and the ependymal layer of the spinal cord 44, 45. These NSCs have the capacity for proliferation, self - renewal, and differentiation into various cell types. Although Endo - NSCs remain quiescent under physiological conditions, they become activated in response to pathological stimuli, such as SCI. However, their activation is limited, and they predominantly differentiate into astrocytes, and to a lesser extent, oligodendrocytes, rather than neurons 46. Notably, Llorens - Bobadilla et al. identified a latent lineage of Endo - NSCs capable of contributing to oligodendrocyte replacement 44.

#### Scar formation

Scar formation is a crucial aspect of the pathology of SCI, persisting throughout the entire pathophysiological process. During the acute phase, astrocytes polarize into distinct A1 and A2 phenotypes, each associated with specific roles in the injury response 47. Concurrently, pericytes derived from blood vessels migrate toward the injury epicenter 48, 49. As the injury progresses into the subacute phase, a scar begins to form, comprising astrocytes derived from resident astrocytes, OPCs, and NSCs. Fibroblast - derived pericytes are essential for scar consolidation during this stage 50. In the chronic phase, the scar stabilizes, effectively limiting inflammation but concurrently inhibiting axonal regeneration 51, 52. The scar consists of both fibrous and glial components. The fibrous portion contains stromal cells at the scar's core, derived from vascular - associated type A pericytes, while the glial portion comprises astrocytes originating from self - replicating astrocytes and endogenous NSCs. Astrocytes derived from endogenous NSCs contribute to strengthening the glial scar. Additionally, the scar includes microglia, macrophages, and an extracellular matrix, with the latter primarily consisting of chondroitin sulfate proteoglycans (CSPGs), which are known to inhibit axonal regeneration 53. While the scar functions as a physical and molecular barrier to restrict the spread of inflammation, its excessive formation significantly impedes axonal outgrowth and functional recovery.

### Cellular imbalance

**Figure 2:**
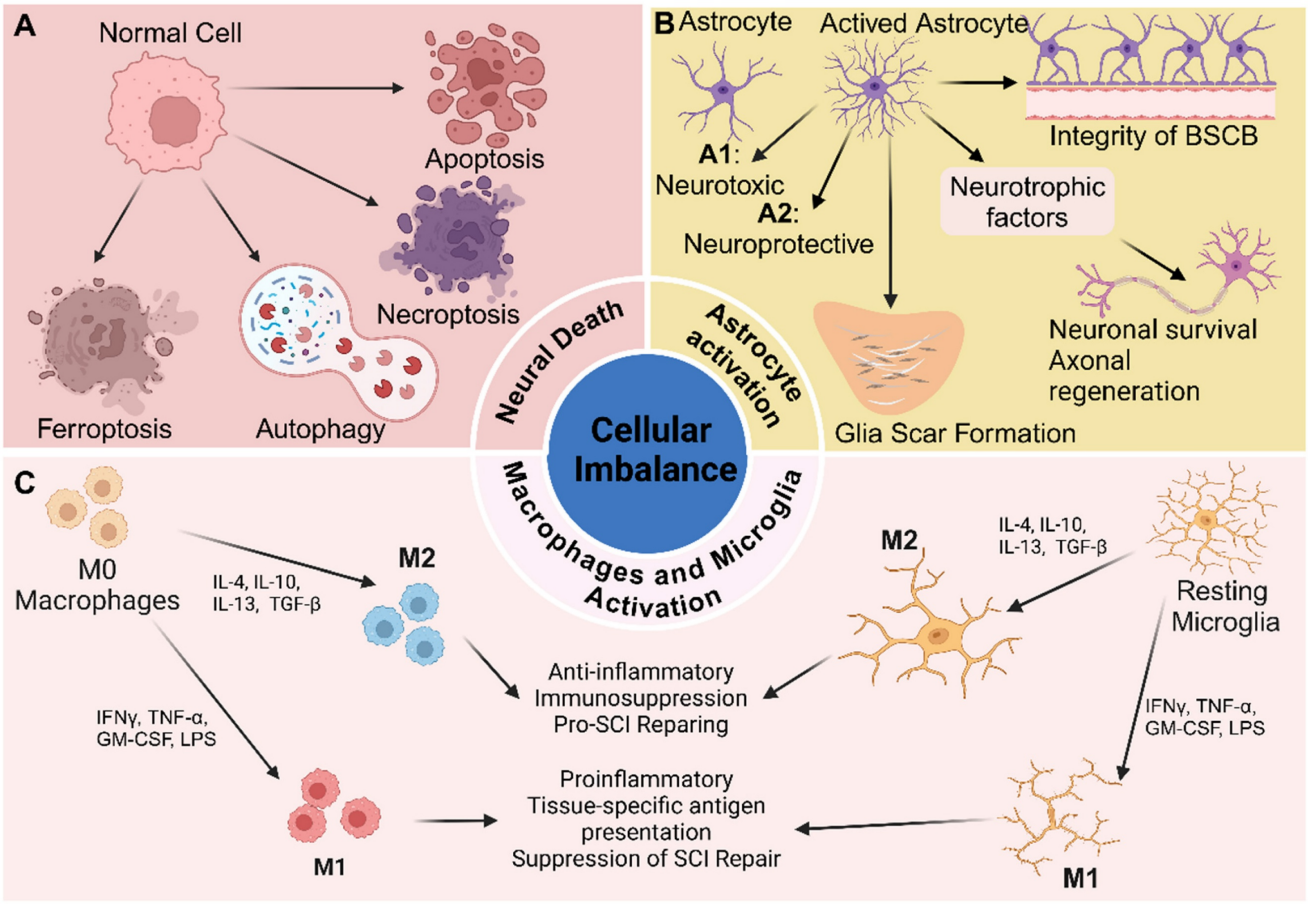
Cellular imbalance of microenvironment after SCI, including neural death, activation of macrophages and microglia, and astrocyte reaction and proliferation. Created with BioRender.com.

Cellular imbalance after SCI denotes the disturbance of the spinal cord's cellular homeostasis following injury, characterized by the activation of macrophages and microglia, reactive astrogliosis and proliferation, and neuronal loss (**Figure 2**).

#### Activation of macrophages and microglia

The infiltration of inflammatory cells following SCI is a key contributor to secondary injury mechanisms, intensifying the initial trauma and impeding recovery. This inflammatory response is characterized by the recruitment of various immune cells, including neutrophils, macrophages, lymphocytes, and microglia, each with distinct roles in mediating both tissue damage and repair 54. Among these cells, macrophages and microglia have garnered particular attention due to their prominent roles in orchestrating both injury progression and regenerative processes (**Figure 2**).

As previously mentioned, disruption of the BSCB following SCI permits peripheral circulating blood to enter the lesion site. Macrophages subsequently infiltrate the area in response to this breach 55. Stimulated by microglia, macrophages from the peripheral circulation begin to accumulate in the injured region approximately 2 - 3 days post - injury. Their numbers peak between 7 and 10 days after SCI and can persist in the lesion for up to 42 days. These cells play a critical role in clearing cellular debris and promoting tissue repair by releasing growth factors and cytokines that support regenerative processes 56. However, dysregulation of macrophage activity, particularly prolonged pro - inflammatory responses, can exacerbate tissue damage and impair recovery, highlighting their dual role in both repair and secondary injury following SCI. 57.

Microglia are the resident immune cells of the CNS, acting as primary immune sentinels in the brain and spinal cord. Under normal physiological conditions, microglia remain relatively quiescent, but are rapidly activated and play central roles in the immune response following SCI 58. Stimulated by cytokines and other signaling molecules, such as IL - 1β, TNF - α, and growth factors secreted by astrocytes and other injured cells, microglia become activated, resulting in enhanced microglial proliferation. The number of activated microglia rises sharply within the first day post - injury, continues to increase over the subsequent week, and plateaus between two to four weeks 59. This sustained activation is essential for clearing cellular debris and damaged cells, thereby contributing to tissue repair. Additionally, microglial activation plays a protective role by limiting the expansion of the lesion site. However, excessive or chronic activation can exacerbate inflammation and secondary injury, underscoring their dual role in mediating both tissue damage and repair.

The polarization of macrophages and microglia is currently an area of intense research interest in the context of SCI. These immune cells can adopt two major polarization states: M1 and M2. The balance between M1 and M2 macrophages/microglia is essential for regulating immune homeostasis 60, 61. To some extent, the ratio of M1 to M2 subtypes influences the balance of the local microenvironment 62. M1 macrophages/microglia are the dominant subtype during the acute phase of SCI. They are recruited to the injury site within the first few days and are activated by signals from damaged tissue and pro - inflammatory cytokines, thereby exacerbating neuroinflammation and neuronal injury. Prolonged dominance of M1 macrophages/microglia ultimately impedes SCI recovery 59. In contrast, M2 macrophages/microglia typically emerge later in the healing process and are associated with tissue repair and the resolution of inflammation. M2 macrophages are anti - inflammatory in nature and contribute to tissue regeneration by producing high levels of IL - 10 and TGF - β, showing impaired activation of the NF - κB pathway, upregulating arginase 1, and downregulating pro - inflammatory cytokines 63-66. Similarly, M2 - polarized microglia promote neuroprotection and recovery following CNS injury by releasing anti - inflammatory cytokines and neurotrophic factors that support neuronal survival, remyelination, and synaptic plasticity 67.

In summary, developing therapeutic strategies to promote tissue repair and mitigate the adverse effects of chronic inflammation requires a thorough understanding of the mechanisms underlying macrophage/microglial polarization and the dynamic balance between M1 and M2 states. Harnessing the beneficial effects of M2 polarization may enhance recovery and improve outcomes across various pathological conditions.

#### Neuronal death

Neurons are essential components of the spinal cord, and neuronal loss is a primary contributor to the poor functional recovery observed after SCI. Accumulating evidence indicates that the mechanisms underlying neuronal loss include apoptosis, necroptosis, autophagy, and ferroptosis 68. Necrosis, in contrast to these programmed forms of cell death, is a passive and uncontrolled process that occurs in a non - programmed manner. During the primary injury phase, direct mechanical trauma causes neurons at the injury site to undergo necrosis 69. In the subsequent secondary injury phase, necrotic cells induce several pathogenic changes, including swelling and deformation of surrounding neurons, altered membrane permeability, and ultimately rupture - a process consistent with necrosis 70. The release of intracellular contents following necrosis triggers inflammatory responses, resulting in widespread neuronal necrosis during the subacute phase 71. As necrosis exacerbates pathological changes, it perpetuates the cycle of neuronal damage. Furthermore, substances released from necrotic cells, such as cytokines and damage - associated molecular patterns (DAMPs), may serve as signals that initiate additional forms of cell death 72.

Apoptosis is a coordinated, energy - dependent, and genetically regulated form of cell death in which caspases serve as the primary executioners. It is a prominent feature of neural tissue following SCI, occurring in nearly all major neural cell types, including neurons, astrocytes, oligodendrocytes, and microglia 73. During the weeks - long period of neuronal apoptosis post - injury, glial cells undergo apoptosis to a greater extent than neurons. The mechanisms underlying neuronal apoptosis involve three major pathways: the extrinsic pathway, activated by the binding of ligands to death receptors in the tumor necrosis factor (TNF) receptor family (including TNF receptor 1, Fas, Fas ligand, p75, and DR3); the intrinsic, or mitochondrial, pathway; and the endoplasmic reticulum (ER) stress pathway 74-76.

Autophagy is a cellular process responsible for degrading and recycling damaged organelles, misfolded proteins, and other intracellular components. This process involves initiation, autophagosome formation, fusion with lysosomes, and subsequent degradation 77, 78. Following SCI, the ratio of LC3 - II to LC3 - I, a commonly used marker for autophagy, rises significantly at 3 days post - injury, peaks at 7 days, and then declines sharply by day 21. However, the role of autophagy in traumatic SCI remains controversial 73. It is widely acknowledged that autophagy plays a dual role: it can promote cell survival by clearing damaged cellular components, yet excessive autophagy may contribute to cell death. Modulating autophagy represents a promising therapeutic strategy for reducing secondary damage and enhancing functional recovery in SCI 79.

Ferroptosis, first identified in 2012 as an iron - dependent, non - apoptotic form of cell death, plays a critical role in neuronal loss following SCI 80. It is characterized by iron accumulation and lipid peroxidation, and is distinctly dependent on ROS 81. After SCI, ferroptosis emerges as a key pathological process, marked by elevated iron levels, ROS buildup, and excessive lipid peroxidation at the injury site. Studies using adult mouse models have demonstrated that conditional ablation of GPX4 in neurons can induce motor neuron degeneration, underscoring the pivotal role of ferroptosis in neuronal cell death 82. Notably, excitotoxicity often precedes the onset of ferroptosis, triggered by glutamate accumulation and the failure of astrocyte - mediated glutamate reuptake. Together, these processes contribute significantly to secondary injury after SCI 83. Despite these insights, the precise mechanisms and broader implications of ferroptosis in SCI remain incompletely understood. Therefore, further investigation is required to elucidate its role in neuronal degeneration and identify potential therapeutic targets.

#### Astrocyte reaction and proliferation

Astrocytes, star - shaped glial cells in the CNS, perform a range of essential functions. These include maintaining the integrity of the blood - brain barrier, regulating neurotransmitter levels, providing metabolic support to neurons, and responding to injury 84, 85. Astrocytes play a pivotal role in maintaining CNS homeostasis and are vital for neuronal function and survival. They supply neurons with energy and neurotransmitters and serve as structural barriers between synapses of adjacent neurons 86.

Following SCI, astrocytes undergo a process termed reactive astrogliosis, characterized by both morphological and functional changes 87. Multiple factors can activate astrocytes, including the release of DAMPs from neurons and glial cells, pro - inflammatory cytokines from activated microglia and infiltrating immune cells, extracellular matrix remodeling, and ionic imbalances. Activated astrocytes can exert neuroprotective effects by secreting neurotrophic factors such as brain - derived neurotrophic factor (BDNF) and nerve growth factor (NGF), which support neuronal survival and promote axonal regeneration. They also contribute to restoring the integrity of the blood - spinal cord barrier after injury. Moreover, by regulating ion and neurotransmitter levels, astrocytes help reestablish homeostasis in the injured spinal cord - an essential aspect of recovery 88, 89. However, while reactive astrogliosis is part of the reparative process, it leads to the formation of a glial scar. This scar can inhibit axonal regeneration by forming a physical barrier and secreting inhibitory extracellular matrix components, such as chondroitin sulfate proteoglycans, which restrict neuronal growth and limit functional recovery 90. In the context of inflammation, astrocytes exhibit a dual role: they can modulate the immune response by releasing anti - inflammatory cytokines, yet excessive activation may foster a pro - inflammatory environment that exacerbates secondary injury.

In summary, astrocytes undergo substantial changes in response to SCI, which may exert both protective and detrimental effects. To develop effective therapeutic strategies that enhance recovery and mitigate the adverse consequences of reactive astrogliosis, a comprehensive understanding of astrocyte function in SCI is essential. To improve functional outcomes, it is crucial to balance astrocyte - mediated neuroprotection with the inhibitory effects of glial scar formation.

### Molecular imbalance

**Figure 3:**
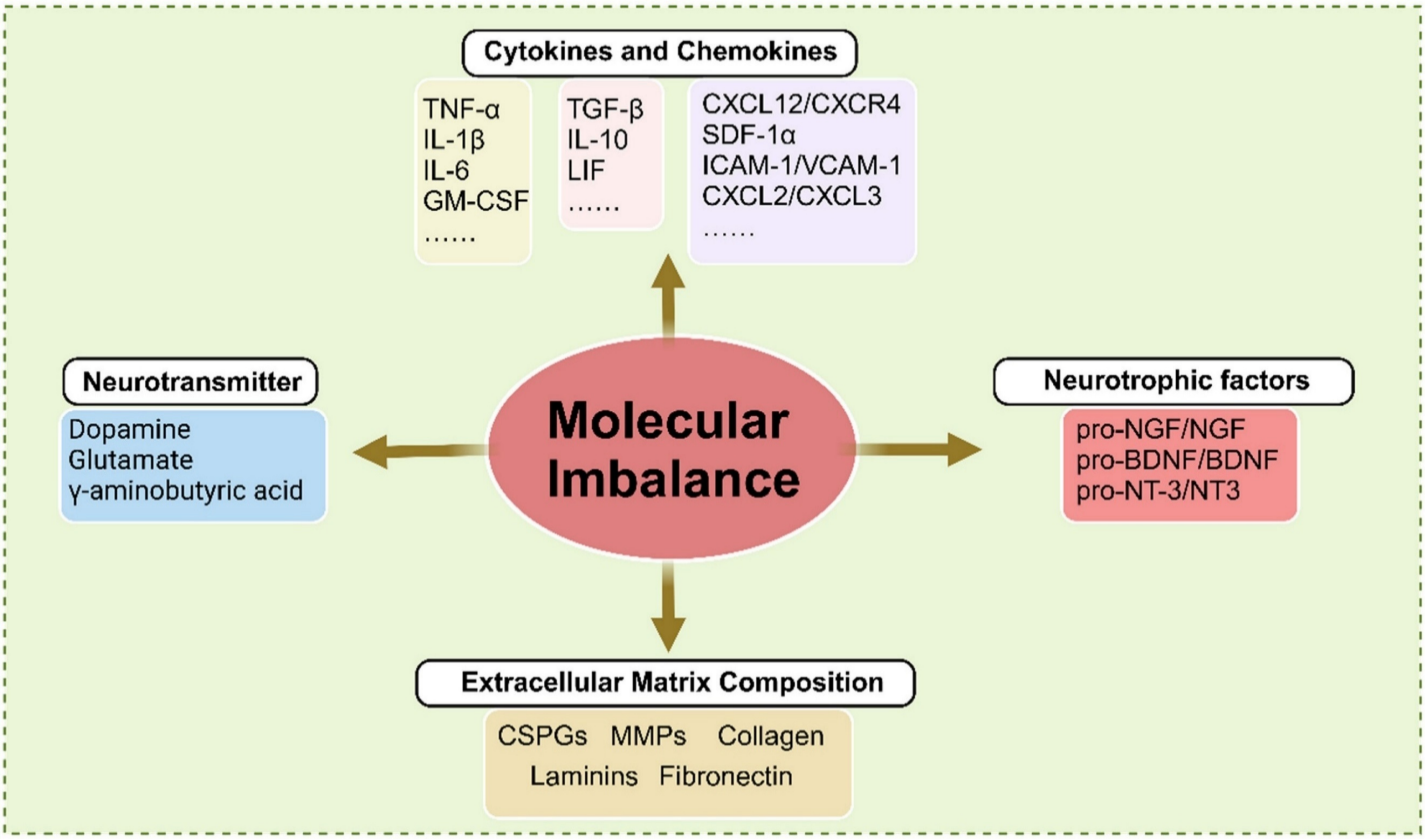
Molecular imbalance of microenvironment after SCI, including cytokines and chemokine, neurotrophic factor, neurotransmitter, and extracellular matrix composition. TNF - α, Tumor necrosis factor - α. IL, Interleukin. GM - CSF, Granulocyte - macrophage colony stimulating factor. ILF, Leukocyte inhibitory factor. CXCL, C - X - C motif ligand. CXCR, C - X - C chemokine receptor. SDF - 1α, stromal cell - derived factor 1α. ICAM - 1, Intercellular adhesion molecule - 1. VCAM - 1, Vascular cell adhesion protein - 1. NGF, Nerve growth factor. Brain - derived neurotrophic factor. NT - 3, Neurotrophin - 3. CSPGs, Chondroitin sulfate proteoglycans. MMPs, Matrix metalloproteinases. Created with BioRender.com.

Molecular imbalance plays a critical role in the pathophysiology of SCI, influencing inflammation, cell death, and regeneration. Following SCI, key molecular alterations include an imbalance in inflammatory mediators and chemokines, altered neurotransmitter levels, dysregulation of neurotrophic factors, and remodeling of ECM components (**Figure 3**).

#### Inflammatory mediators and chemokines imbalance

After SCI, activated microglia, macrophages, and astrocytes secrete inflammatory mediators such as TNF - α, IL - 1β, and IL - 6, which increase rapidly. Inflammation functions as a double - edged sword: while appropriate inflammatory responses protect neural tissue and help contain damage, excessive and prolonged release of pro - inflammatory cytokines can exacerbate neuronal injury. These cytokines promote neuronal apoptosis and sustain inflammation, thereby impeding recovery 91. In contrast, anti - inflammatory cytokines such as IL - 10 and TGF - β are essential for resolving inflammation and facilitating tissue repair. Specifically, IL - 10 inhibits the production of pro - inflammatory cytokines, creating a more favorable environment for recovery 92. Research has shown that increasing the expression of anti - inflammatory cytokines may enhance functional outcomes following SCI.

Concurrently, chemokines such as C - C motif ligand 2 (CCL2) and C - X - C motif ligand 1 (CXCL1) are upregulated following SCI. This upregulation attracts immune cells, including neutrophils and macrophages, to the injury site, which subsequently infiltrate the disrupted BSCB 93. Research has indicated that different chemokines exhibit distinct spatial and temporal expression profiles after SCI. For instance, CXCL2 and CXCL3 are rapidly upregulated within hours of injury, promoting the recruitment of monocytes and T cells to the damaged area 93, 94. Initially, the influx of immune cells is beneficial, as it facilitates debris clearance and the secretion of neurotrophic factors. However, the prolonged presence of inflammatory cells can lead to secondary damage, underscoring the dual role of chemokines in SCI pathophysiology.

Furthermore, specific chemokines such as CXCL12 are involved in promoting neuronal survival and regeneration. CXCL12 has been shown to enhance the migration of neural precursor cells, indicating its therapeutic potential for facilitating repair 95. In contrast, excessive expression of pro - inflammatory chemokines can exacerbate tissue damage and hinder recovery. Consequently, maintaining a balance between the protective and detrimental effects of chemokines is critical in determining the overall outcome of SCI. Understanding the roles of chemokines and cytokines in SCI offers promising avenues for therapeutic intervention. By targeting specific cytokines or chemokines, or their associated signaling pathways, it is possible to modulate the inflammatory response, reduce secondary damage, and promote functional recovery. Therapeutic strategies such as neutralizing antibodies and small - molecule inhibitors have demonstrated potential in preclinical studies, showing promise in enhancing functional recovery in individuals with SCI.

#### Neurotransmitter levels change

Neurotransmitters are chemical messengers that facilitate signal transmission across synapses, enabling communication between neurons or from neurons to other target cells, such as muscles and glands. These molecules play a critical role in spinal cord function by mediating interactions between neural circuits and peripheral tissues, including muscles 96. When an action potential reaches the presynaptic terminal of a neuron, it triggers the release of neurotransmitters into the synaptic cleft. These neurotransmitters then bind to specific receptors on the postsynaptic membrane, inducing excitatory or inhibitory responses that regulate postsynaptic cellular activity. This precise regulation of synaptic transmission is essential for coordinating motor and sensory functions and for maintaining homeostasis within the nervous system 97, 98.

Following SCI, neurotransmitter levels undergo significant changes, influencing both the immediate response to injury and long - term recovery. Glutamate, a major excitatory neurotransmitter essential for synaptic plasticity and cognitive processes such as learning and memory, is excessively released after SCI 96. Elevated glutamate levels can induce excitotoxicity, whereby overstimulation of glutamate receptors, particularly NMDA receptors, leads to neuronal injury and death, contributing to secondary damage and worsening injury to surrounding tissues. GABA, the primary inhibitory neurotransmitter, may also be dysregulated due to the disrupted balance between excitatory and inhibitory signaling following SCI 99. This imbalance, often manifested as reduced GABAergic signaling, increases spinal neuron excitability and contributes to neuropathic pain and hyperreflexia. Ultimately, this disruption complicates recovery and may lead to chronic pain syndromes.

In conclusion, neurotransmitters are essential chemical messengers that regulate numerous physiological processes and play a central role in neural communication. Maintaining their proper balance and function is critical for preserving synaptic integrity and neural network stability. Therefore, understanding neurotransmitter alterations following spinal cord injury is crucial for developing targeted therapeutic strategies aimed at mitigating their detrimental effects and facilitating functional recovery.

#### Alteration of neurotrophic factors

Neurotrophic factors, also known as neurotrophins, constitute a family of proteins that play critical roles in the growth, survival, development, and maintenance of neurons within the nervous system. Members include NGF, BDNF, GDNF, neurotrophin - 3 (NT - 3), and neurotrophin - 4/5 (NT - 4/5). These proteins bind to tropomyosin - related kinase (Trk) receptors and the low - affinity neurotrophin receptor p75. By promoting synaptic plasticity, regeneration, and repair, they support overall neuronal health 100, 101. During development, neurotrophic factors are essential for guiding neural cells and ensuring the proper formation of neural circuits.

Neurotrophic factors (NFs) play a crucial role in promoting the survival and proliferation of various neural cell types, as well as supporting axonal regeneration following SCI. Enhancing neurotrophic factor signaling can facilitate neuronal differentiation, survival, axonal growth, and synaptic plasticity in the context of SCI treatment 100-103. However, NFs are initially synthesized as precursors, some of which function as distinct ligands that induce cell death. Multiple studies have shown that the pro - neurotrophin forms of NGF, BDNF, and NT - 3 are present after SCI and contribute to neural cell death. This results in a relative deficiency of mature NFs, thereby disrupting the balance of the neurotrophic microenvironment. For example, pro - NGF has been shown to induce oligodendrocyte death by activating the p75 receptor, which compromises myelin sheath integrity 104. Additionally, pro - BDNF increases within 1 to 3 days after SCI and suppresses macrophage migration and infiltration 105. Several studies have demonstrated that reducing pro - neurotrophin levels improves outcomes in SCI models, providing strong evidence of their detrimental role in the injury response.

#### Extracellular Matrix Changes

ECM of the spinal cord is a complex macromolecular network composed of proteins, glycoproteins, and polysaccharides. It provides structural support and regulates various cellular activities 106. Among its components, collagens are the most abundant structural proteins, forming the backbone of the ECM framework and conferring tensile strength. CSPGs, the primary proteoglycans in spinal cord tissue, modulate cell adhesion, migration, and axonal growth and are key inhibitors of regeneration after injury 107. As representative glycoproteins, laminin and fibronectin support cell attachment and migration. Additionally, elastin and matrix metalloproteinases (MMPs) are critical ECM components. Elastin contributes to maintaining tissue elasticity and flexibility, while MMPs facilitate the degradation and remodeling of ECM constituents 108. The ECM serves multiple essential functions in the spinal cord, including providing mechanical support, regulating cell - cell and cell - matrix interactions, guiding neural development, and maintaining tissue homeostasis. It acts as a scaffold for cellular organization and influences axon guidance, synaptic plasticity, and neuronal survival.

Following SCI, significant alterations occur in the composition of the ECM, influencing both the acute damage response and long - term recovery. Reactive astrocytes become the primary source of CSPGs, whose levels increase substantially, particularly in the perilesional region. In adult mammals, CSPGs signal through two major receptor protein tyrosine phosphatases (RPTPs): protein tyrosine phosphatase sigma (PTPσ) and the leukocyte antigen - related (LAR) subfamily 109. Through these receptors, CSPGs inhibit axonal regeneration by activating the RhoA/ROCK and PKC signaling pathways 110. Moreover, CSPGs interfere with autophagy by suppressing autophagosome - lysosome fusion, thereby impairing autophagic regulation at the axonal growth cone. Inhibition of CSPG - PTPσ signaling has been shown to restore autophagic flux, promote axonal and synaptic reorganization, preserve remaining motor neurons, and improve functional recovery after SCI 111. CSPGs also disrupt immune modulation by blocking the Toll - like receptor 4 (TLR4) - dependent transition of immune cells from a pro - inflammatory to a pro - repair phenotype, highlighting another mechanism of secondary injury 112. In parallel, collagen levels also undergo dynamic changes following SCI. In the early phase, increased activity of MMPs accelerates collagen degradation, contributing to tissue breakdown and exacerbating inflammation. This degradation compromises spinal cord structural integrity and impairs the ability of axons to regenerate. During the chronic phase, collagen accumulates as part of the glial scar, forming a physical barrier that inhibits axonal regrowth.

Molecular imbalance following SCI arises from the complex interplay among inflammatory mediators, altered neurotransmitter levels, and changes in growth factors and ECM components. Understanding these molecular changes provides critical insights into the mechanisms underlying secondary injury and recovery. Targeting these molecular pathways may provide novel therapeutic opportunities to enhance recovery, promote neuronal regeneration, and improve functional outcomes after SCI.

## History, biogenesis and structure of exosomes

Exosomes are small extracellular vesicles secreted by numerous cell types, playing a key role in mediating intercellular communication. These vesicles typically range from 30 - 150 nanometers in diameter. They are released into the extracellular space and can be detected in various biological fluids, such as blood, urine, and saliva 19, 113.

### History of exosomes - based SCI therapies

Exosomes, a subtype of extracellular vesicles (EVs), have emerged as key mediators of intercellular communication. However, their recognition and characterization have evolved gradually over time. The earliest observations of extracellular particles date back to 1877, when Edmunds first identified serum-derived particles using dark-field illumination. These particles were later identified as lipid - rich structures in 1939, yet were initially dismissed as “blood dust” due to the lack of functional understanding. By 1962, advancements in microscopy enabled clearer visualization of vesicular structures within cells, though their physiological significance remained speculative 114.

A pivotal shift occurred in 1969 when matrix vesicles were implicated in cartilage calcification, indicating that extracellular vesicles might play active physiological roles 115. The term exosome was introduced in 1981 to describe vesicles measuring 50 - 1,000 nm in diameter 116. In 1983, independent studies by Stahl and Johnstone demonstrated that vesicles released by maturing reticulocytes could fuse with the plasma membrane and discharge their cargo via exocytosis, marking the first functional insight into exosome - mediated molecular transfer 117. This finding was further supported by electron microscopy in 1985, and in 1987, the biogenesis of exosomes via inward budding of multivesicular endosomes (MVEs) was formally described 118. Nevertheless, their biological functions remained largely undefined until 1996, when Raposo et al. showed that B cell - derived exosomes enriched in MHC class II molecules could activate T cells, thereby establishing exosomes as mediators of immune communication and stimulating broader interest in their signaling roles 119. In subsequent years, a variety of mammalian cells were found to secrete exosomes into the extracellular milieu 120. A notable breakthrough came in 2007, when Valadi et al. revealed that exosomes derived from human and murine mast cells carry abundant mRNAs and microRNAs, which can be transferred to recipient cells and translated into functional proteins - further solidifying the role of exosomes in horizontal gene regulation and intercellular signaling 121.

Since the early 21st century, exosomes have garnered increasing attention as promising therapeutic tools for neurological diseases. This interest has been fueled by growing evidence of their roles in immunoregulation and tumor biology, leading researchers to investigate their relevance in the nervous system. In 2006, Fauré et al. provided the first evidence that primary neurons release exosomes in response to depolarization, thereby introducing exosomes into the field of neurobiology 122. The 2013 Nobel Prize in Physiology and Medicine, awarded for discoveries in vesicular transport regulation, further underscored the foundational relevance of vesicle biology to exosome research, which has generated great interest among researchers in the study and citation of exosomes 123. Subsequent studies demonstrated that exosomes released from activated glutamatergic synapses preferentially bind to neurons, supporting the concept of exosome-mediated interneuronal communication 124. In 2015, it was reported that neuron - derived exosomes could deliver small interfering RNA (siRNA) into the CNS, resulting in reduced inflammasome activation after injury 125. In 2017, Huang et al. demonstrated for the first time that systemic administration of MSC - derived exosomes attenuated neuronal apoptosis and inflammation, promoted angiogenesis, and improved functional recovery following SCI 126.

**Figure 4:**
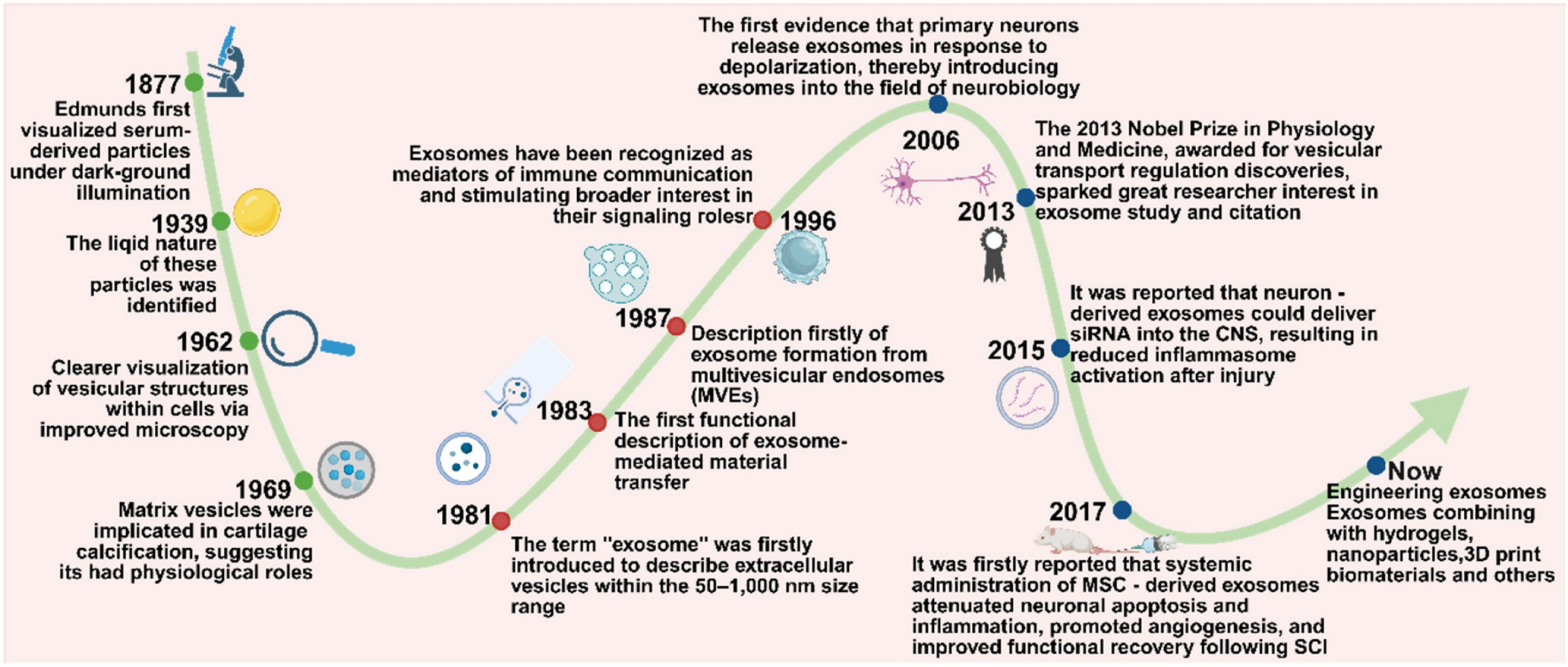
A timeline of landmark studies on exosome-based SCI therapies. Created with BioRender.com. Created with BioRender.com.

Since then, the application of exosomes in SCI has progressed considerably. Recent research has focused on engineering exosomes and combining them with biomaterials - such as hydrogels, nanoparticles, and 3D print biomaterials - to enhance targeted delivery, stability, and therapeutic efficacy in spinal cord repair. Over the past decade, exosome - based therapy has rapidly gained momentum. Exosomes are now recognized for their multifaceted roles in reinforcing the BSCB, promoting axonal regeneration, facilitating angiogenesis, mitigating neuronal apoptosis, and modulating immune responses. Owing to their intrinsic cargo of nucleic acids and regulatory proteins, exosomes have become promising candidates for clinical translation, serving as bioactive vehicles for therapeutic interventions in SCI **(Figure 4)**.

### Biogenesis of exosomes

Exosomes biogenesis is tightly regulated at the cellular level and generally consists of three key steps: the formation of early endosomes, maturation into multivesicular bodies (MVBs), and the eventual release of exosomes 127. In the initial step, the plasma membrane invaginates to form a cup - shaped structure that encloses soluble extracellular proteins and membrane - bound proteins, resulting in the formation of an early - sorting endosome (ESE) 128. The endoplasmic reticulum and trans - Golgi network may also contribute to the development and molecular composition of the ESE. Subsequently, ESEs mature into late - sorting endosomes (LSEs), which then develop into MVBs, also referred to as multivesicular endosomes. MVBs arise through the inward budding of the endosomal limiting membrane, resulting in the formation of intraluminal vesicles (ILVs), the precursors of exosomes. MVBs may either fuse with the plasma membrane to release ILVs as exosomes or fuse with lysosomes or autophagosomes for degradation 129. This tightly controlled process is regulated by a variety of molecules, including endosomal sorting complexes required for transport ESCRT), ALG - 2 - interacting protein X (Alix), tumor susceptibility gene 101 (TSG101), Rab GTPases, tetraspanins, and heat shock proteins (HSPs), among others 130.

**Figure 5:**
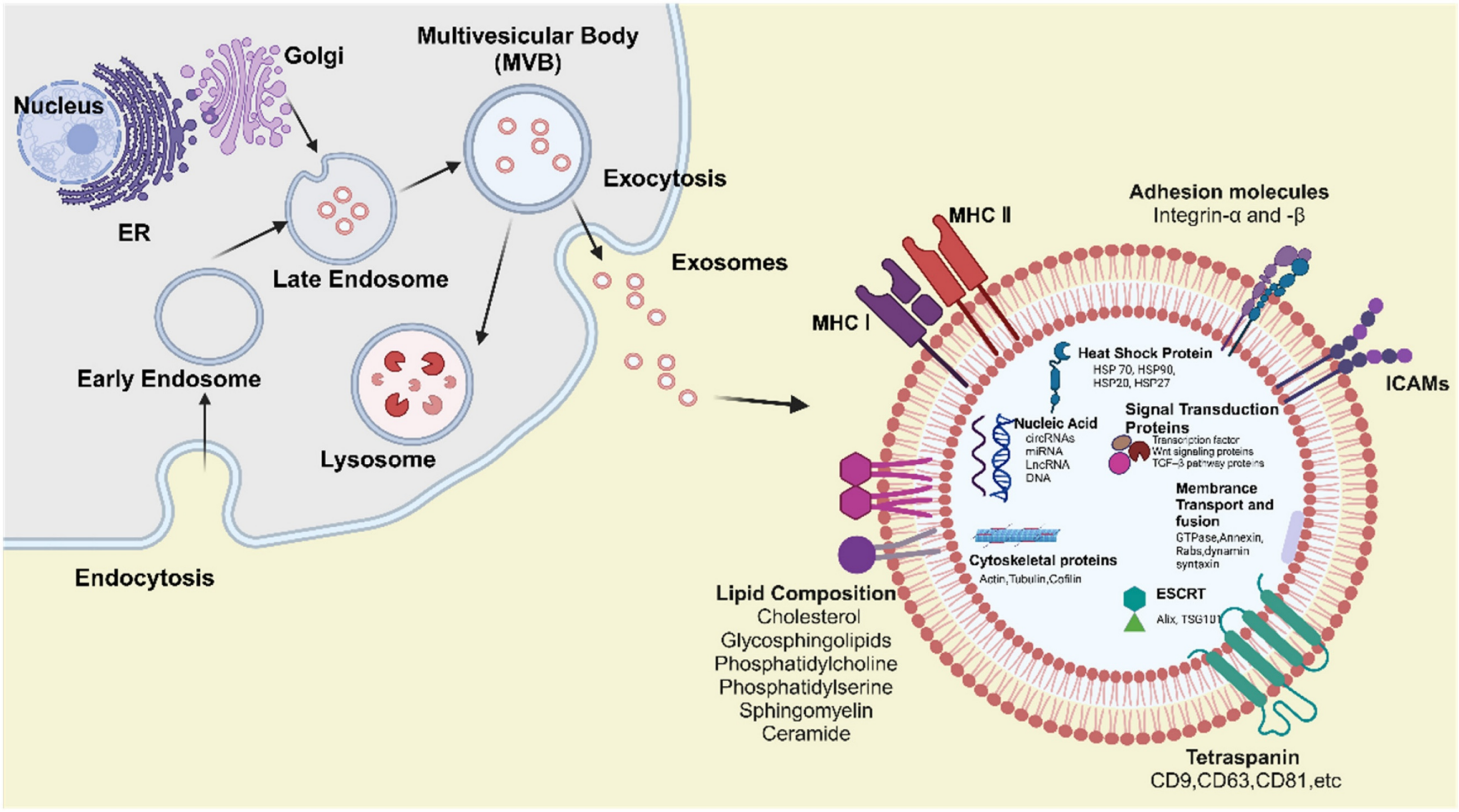
The biogenesis, structure and composition of exosomes. Created with BioRender.com.

Upon release, exosomes can spread to adjacent tissues and organs or enter the systemic circulation, thereby reaching distant sites within the body. Recipient cells primarily internalize exosomes via three mechanisms: endocytosis, direct fusion with the plasma membrane, and ligand - receptor interactions 131. It is hypothesized that once internalized, exosomes undergo back - fusion with the membranes of MVBs, releasing their functional cargo, such as RNAs, DNAs, and proteins, into the cytoplasm of recipient cells 20. This cargo can subsequently regulate cellular processes, enabling exosomes to play a critical role in modulating physiological and pathological activities throughout the body (**Figure 5**).

### Structure and composition of exosomes

Exosomes are small extracellular vesicles enclosed by a lipid bilayer membrane and are derived from the endosomal membrane during their formation. They are enriched with specific proteins, lipids, enzymes, and nucleic acids. Notably, the composition and biological function of exosomes are highly dynamic and influenced by the cellular origin, physiological status, and external stimuli such as hypoxia or oxidative stress, leading to significant heterogeneity among exosomal populations 19, 132. These components enable exosomes to contribute to intercellular communication by mediating the transfer of signaling molecules to both local and distant target cells (**Figure 5**).

#### Lipid composition

The lipid composition of exosomes differs from that of the plasma membrane of the parent cell, partly due to their enrichment with lipids originating from Golgi and endosomal membranes. These include cholesterol, glycosphingolipids, phosphatidylcholine, phosphatidylserine, sphingomyelin, and ceramide 133, 134. These lipids contribute to the distinct rigidity of exosomes and play crucial roles in their biogenesis, structural integrity, and biological function. Phosphatidylserine is typically located on the inner leaflet of the exosome membrane; however, it may become externalized during exosome release. This externalization facilitates recognition and binding to recipient cells via specific receptors 135. Phosphatidylcholine, a major constituent of the exosomal membrane, contributes to maintaining membrane fluidity and bilayer stability 136. Ceramide is essential for exosome biogenesis, particularly in the formation of ILVs within MVBs, by promoting membrane curvature and vesicle budding through ESCRT - independent mechanisms 137. Sphingomyelin and glycosphingolipids function as bioactive signaling molecules, capable of modulating cell survival, apoptosis, and immune responses, thereby influencing the behavior of both donor and recipient cells 138.

#### Proteins composition

Exosomes contain a variety of proteins that are essential for their structure, biogenesis, and functions - including intercellular communication. These proteins are generally classified into membrane - associated proteins and internal (cytosolic) proteins. Membrane - associated proteins can be further categorized into four major groups: tetraspanins, transport and fusion proteins, adhesion molecules, and heat shock proteins (HSPs) 139-141. Tetraspanins, such as CD9, CD63, and CD81, are highly expressed on exosomes derived from most cell types. They are involved in exosome formation, membrane organization, and cell targeting 142. Interestingly, emerging evidence indicates that external conditions, such as hypoxia, can selectively enrich specific sets of tetraspanins and adhesion molecules in exosomes, thereby enhancing their targeting efficiency and altering their uptake by recipient cells 132, 143. Moreover, tetraspanins promote exosome-cell interactions and play essential roles in the binding and uptake of exosomes by recipient cells via ESCRT - independent pathways. These proteins are commonly used as exosomal markers due to their enrichment in exosomes and low abundance in other vesicle types. Alix and TSG101, key transport and fusion proteins, are integral to the formation of ILVs within MVBs, as components of the ESCRT complex responsible for cargo sorting and exosome release 144. Integrins and intercellular adhesion molecules (ICAMs) facilitate the binding of exosomes to specific receptors on recipient cells, thereby mediating intercellular communication. Notably, integrins also contribute to tissue - specific targeting of exosomes 145. Within exosomes, HSPs such as HSP70 and HSP90 protect cargo proteins during transit and participate in immune modulation by interacting with antigen - presenting cells.

Internal proteins primarily include enzymes, signal transduction proteins, and cytoskeletal proteins 146. These proteins originate from the cytoplasm of the parent cell and are selectively packaged into exosomes through specific sorting mechanisms during the formation of MVBs. The sorting of these proteins is tightly regulated by cellular signaling pathways, including those activated under stress conditions, which ensure the functional specificity of exosomal cargo 19. Exosomes carry various enzymes involved in metabolic processes, which can modulate the metabolic activity of recipient cells and consequently influence their cellular behavior. Some exosomes also contain proteases capable of degrading extracellular matrix components, contributing to physiological and pathological processes such as tissue remodeling and cancer metastasis. Additionally, exosomes are enriched with signal transduction proteins, including kinases, GTPases, transcription factors, receptor tyrosine kinases, adhesion - and migration - related proteins, Wnt signaling components, and proteins involved in the TGF - β signaling pathway 147. These molecules can activate or suppress diverse signaling cascades, thereby modulating intercellular communication and influencing the functional responses of recipient cells.

#### Nucleic acid

In addition to lipids and proteins, nucleic acids represent another major component of exosomal cargo. In 2007, Valadi and colleagues first reported that exosomes derived from mast cells contained both mRNA and microRNA (miRNA), demonstrating that exosome - mediated transfer of these nucleic acids constitutes a novel mechanism of genetic exchange between cells. This discovery opened a new avenue of research and potential applications for exosomes. Subsequent studies confirmed the presence of various mRNAs and small non - coding RNAs, including miRNAs, within exosomes 121. Moreover, RNA sequencing technologies have revealed that exosomes transport diverse types of non - coding RNAs, such as circular RNAs (circRNAs), miRNAs, and long non - coding RNAs (lncRNAs) 148. Recent findings suggest that the RNA cargo of exosomes is selectively packaged through interactions with RNA - binding proteins, and that stress - related conditions such as hypoxia or inflammation can dramatically reshape the exosomal RNA landscape, influencing gene expression patterns in recipient cells 149-151. The mRNA encapsulated within exosomes can be delivered to recipient cells and translated into functional proteins, thereby influencing cellular function and behavior. This process plays a pivotal role in biological contexts such as tissue repair, immune regulation, and tumor progression. miRNAs are short, single - stranded non - coding RNAs (ncRNAs) that typically bind to recognition motifs in the 3′ untranslated region (3′ UTR) of target mRNAs, promoting mRNA degradation and inhibiting gene expression. lncRNAs contribute to the regulation of cell differentiation and the cell cycle. CircRNAs have been proposed as miRNA sponges, competing with miRNAs to regulate gene expression 152.

## Functions and mechanisms of exosomes on SCI

**Figure 6:**
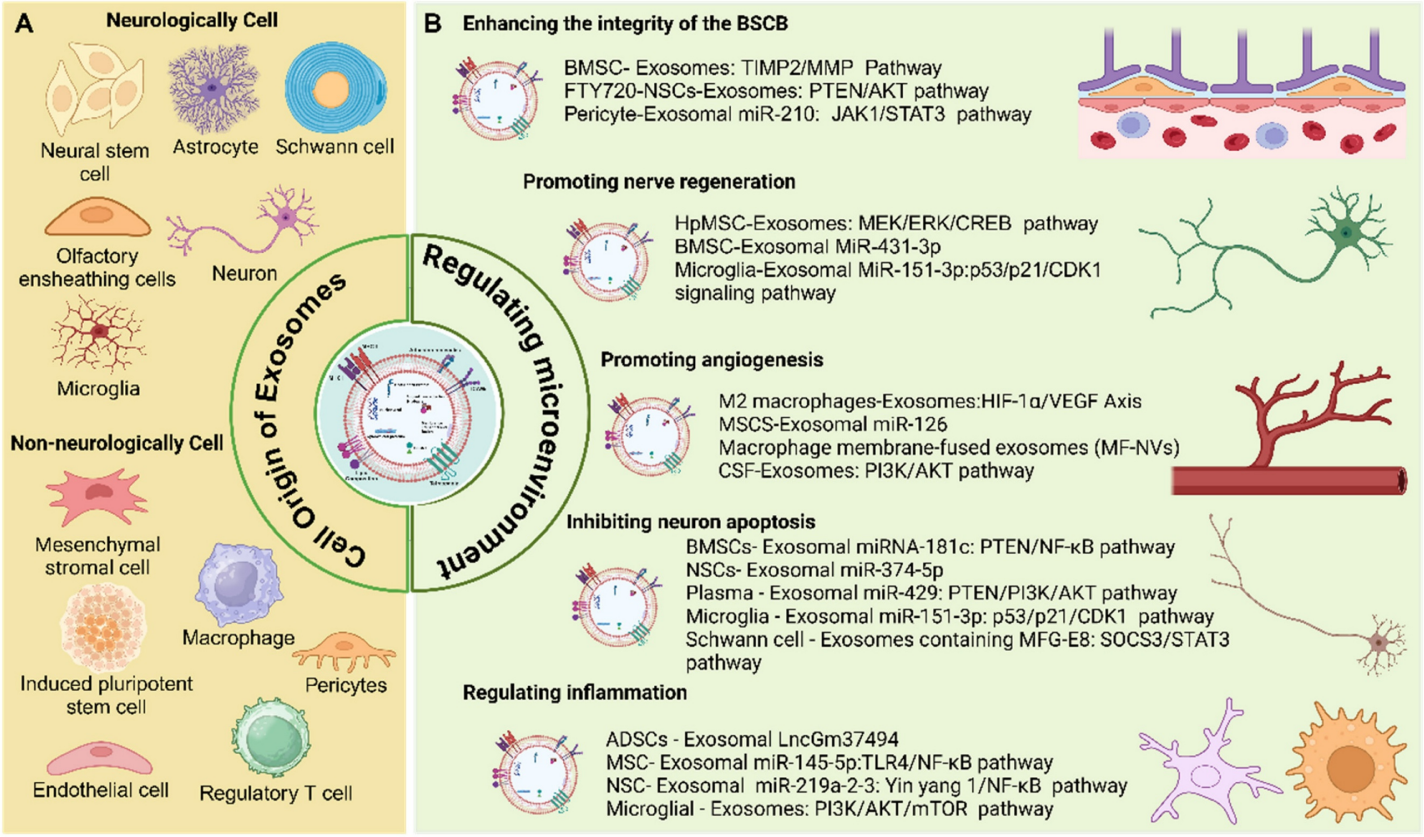
Cell source of exosomes and its mechanism for modulating microenvironment after SCI. Created with BioRender.com.

In SCI, exosomes support multiple neuroprotective and neuroregenerative processes by modulating the injured microenvironment. These mechanisms include suppressing inflammatory responses, enhancing the expression of neurotrophic factors, restoring ECM homeostasis, and modulating astrocyte polarization to limit glial scar formation. In addition, exosomes offer several advantages, including efficient cargo encapsulation and the ability to penetrate the BSCB to reach the site of injury. Due to their therapeutic bioactivity, inherent biocompatibility, and capacity for targeted delivery, exosomes are considered key components of the cellular secretome and hold significant promise as candidates for cell - free therapies. These therapies offer the potential to circumvent challenges associated with cell transplantation, such as immune rejection and uncontrolled cell proliferation or differentiation. This section focuses on the cellular sources of exosomes, their functional roles in SCI, and the underlying mechanisms through which exosome - mediated therapies exert their effects (**Figure 6**).

### Cell source of exosomes and its function on SCI

Various cell types can secrete exosomes with distinct functions, depending on their cellular origin. These functions include regulation of apoptosis, promotion of angiogenesis, facilitation of nerve regeneration, and support of tissue repair. Exosomes are commonly classified as neurologically or non - neurologically derived based on their cellular source. Neurologically derived exosomes include those secreted by neural stem cells, neurons, microglia, astrocytes, Schwann cells, and olfactory ensheathing cells **(Table 1)**. Non - neurologically derived exosomes include exosomes secreted by mesenchymal stromal cells, macrophages, platelet - rich plasma, pericytes, endothelial cells, induced pluripotent stem cells, and regulatory T cells **(Table 2)**.

#### Neurologically derived exosomes

##### Neural stem cell‑derived exosomes

NSCs are multipotent stem cells residing in the CNS, with the ability to self - renew and differentiate into neurons, oligodendrocytes, and astrocytes. After SCI, quiescent NSCs in the spinal cord become activated in response to injury - associated signals such as cytokines and growth factors, particularly in regions near the injury site, such as the ependymal zone surrounding the central canal. However, most activated NSCs preferentially differentiate into astrocytes rather than neurons 175, largely due to the pro - inflammatory microenvironment, which promotes astrocytic differentiation and contributes to glial scar formation, a major physical and chemical barrier to axonal regeneration. Therefore, strategies aimed at promoting neuronal differentiation of NSCs represent a promising approach for SCI therapy. Studies have shown that NSC transplantation, combined with modulation of cell fate, can enhance nerve regeneration 176-178. After transplantation, NSCs not only have the potential to replace damaged neurons but also secrete neuroprotective and immunomodulatory factors, such as BDNF and GDNF 179, 180. However, NSC transplantation faces several significant limitations, including low survival rates, poor BSCB penetration due to their large size, tumorigenic potential, and ethical concerns 181, 182. Increasing evidence supports the therapeutic potential of exosomes derived from NSCs. For instance, exosomes from NSCs undergoing necroptosis have been shown to mediate cellular communication after SCI by upregulating TSC2 in recipient cells 183. NSC - derived exosomes (NSC - Exos) have demonstrated efficacy in SCI repair, including reducing pathological changes, improving hindlimb motor function, alleviating hypoxia, and regulating key molecular pathways such as PTEN/AKT 153, 184, 185. These exosomes improve neuronal morphology, reduce inflammation and edema, downregulate pro - apoptotic markers such as Bax and AQP4, upregulate tight junction proteins like claudin - 5 and anti - apoptotic Bcl - 2, and inhibit neuronal apoptosis. Ma et al. found that NSC - Exos preconditioned with IGF - 1 could promote neuronal regeneration, inhibit apoptosis, and reduce neuroinflammation via miR - 219a - 2 - 3p 154. Zhang et al. reported that NSC - Exos enhanced SCI recovery by activating autophagy through the miR - 374 - 5p/STK4 axis 155. Other studies have demonstrated that these exosomes deliver VEGF - A to spinal cord microvascular endothelial cells (SCMECs), promoting angiogenesis and facilitating neurological recovery 156. Collectively, these findings indicate that NSC - Exos are a novel and promising therapeutic candidate for SCI repair.

##### Neuron - derived exosomes

Neurons are fundamental cellular components of the CNS and play a vital role in its diverse functions. As the primary conduits for electrical and chemical signals, neurons are crucial for processing and transmitting information. Together, they form a complex network that underpins all aspects of brain and spinal cord activity 186. Moreover, neurons contribute to CNS homeostasis by facilitating crosstalk with adjacent astrocytes and microglia 187. Following SCI, neurons suffer significant loss of structural integrity due to mechanical trauma, leading to cell death and functional deficits at the injury site 6, 188. Consequently, numerous studies have aimed to promote neuronal survival or increase neuronal populations as part of SCI treatment strategies. Among these, neuron - derived exosomes (Neuron - Exos) have have garnered increasing interest for their therapeutic potential. Xu et al. reported that Neuron - Exos can reverse microglial and astrocyte activation, promote the maturation of OPCs both *in vivo* and *in vitro*, and enhance neurite outgrowth and neuronal differentiation from neural stem cells 189. Jiang et al. demonstrated that Neuron - Exos are enriched with miR - 124 - 3p, which inhibits the activation of M1 microglia and A1 astrocytes by suppressing myosin heavy chain 9 (MYH9) activity. This, in turn, modulates the PI3K/AKT/NF - κB signaling pathway, thereby promoting functional recovery in murine SCI models 157. Wang, et al. developed an injectable decellularized extracellular matrix hydrogel embedded with cortical Neuron - Exos, which enhanced the local microenvironment, reduced early - stage neuronal apoptosis, stimulated the activation and differentiation of endogenous NSCs, partially suppressed glial scar formation, and synergistically promoted myelinated axon regeneration and locomotor recovery 190.

##### Microglia - derived exosomes

Microglia are specialized immune cells of the central nervous system (CNS), comprising approximately 10-15% of all CNS cells. Unlike other CNS - resident cells derived from neural progenitors, microglia originate from the myeloid lineage and are closely related to peripheral macrophages. However, they remain functionally confined within the CNS throughout their lifespan. As such, microglia play a vital role in maintaining CNS homeostasis. Their functions include immune surveillance, clearance of apoptotic cells, misfolded proteins, and cellular debris, neuroprotection, injury repair, and regulation of inflammatory responses 191, 192.

Following SCI, microglia are among the earliest responders at the lesion site. Upon activation, they release pro - inflammatory cytokines that contribute to the initiation of the inflammatory cascade. While this inflammation facilitates debris clearance, it can also exacerbate secondary tissue damage, leading to an unfavorable microenvironment for neural repair 193. Depending on the nature of external stimuli, activated microglia may exhibit either neuroprotective or neurotoxic phenotypes. They support wound healing by facilitating the formation of glial repair tissue and promoting astrocyte clustering through cytokine signaling, which is essential for astrocyte proliferation and scar formation 194. Furthermore, microglia phagocytose axonal debris and release excessive levels of pro - inflammatory cytokines, which can negatively affect neuronal regeneration. Prolonged microglial activation following SCI has been linked to ongoing neurodegeneration and persistent neurological deficits 195.

Recent studies have highlighted the significant role of exosomes in modulating microglial function and promoting neurorepair following neurotrauma 23, 196-198. For example, Li et al. reported that microglia - derived exosomes (Microglia - Exos) enhance neurological recovery in SCI mice by delivering miR - 151 - 3p to neurons. This microRNA exerts neuroprotective effects by reducing neuronal apoptosis and promoting axonal growth through the p53/p21/CDK1 signaling pathway 162. Similarly, Peng and his colleague demonstrated that Microglia - Exos exert antioxidant effects and positively regulate vascular regeneration and neurological recovery after SCI via activation of the Keap1/Nrf2/HO - 1 signaling pathway 163. Huang et al. reported that elevated levels of miR - 124 - 3p in Microglia - Exos following TBI suppress neuronal inflammation and enhance neurite outgrowth by targeting neurons 199. In the context of TSCI, exosomes derived from M2 - polarized microglia have shown notable therapeutic potential. These exosomes reduce pyroptosis in spinal neurons, support axonal regeneration, and improve functional outcomes by inhibiting the AIM2/ASC/Caspase - 1 signaling pathway 160. Additionally, they attenuate the activation of neurotoxic A1 astrocytes by suppressing NF - κB signaling, thereby preserving spinal tissue and enhancing motor function recovery 159. Furthermore, M2 Microglia - Exos have been shown to facilitate vascular regeneration and neurological repair through OTULIN - mediated activation of the Wnt/β - catenin pathwa 200.

In summary, Microglia - Exos represent promising therapeutic agents for modulating the post - injury microenvironment and supporting functional recovery in SCI.

##### Astrocyte - derived exosomes

Astrocytes, star - shaped glial cells in the CNS, originate from NSCs during development. They play essential roles in maintaining the BBB, regulating cerebral blood flow, and supplying metabolic support to neurons. In addition, astrocytes help maintain neurotransmitter homeostasis by clearing excess neurotransmitters, particularly glutamate, from the synaptic cleft to prevent excitotoxicity. In response to CNS injury, astrocytes undergo a process known as reactive astrogliosis, which can have both protective and detrimental effects on surrounding tissue 201, 202. Reactive astrocytes are further categorized into A1 and A2 phenotypes. A1 astrocytes, typically induced by inflammatory stimuli, exhibit neurotoxic properties and can exacerbate neuronal damage. In contrast, A2 astrocytes are considered neuroprotective, supporting cell survival and promoting tissue repair following injury 203, 204. Representing a double - edged sword, reactive astrocytes also contribute to glial scar formation. Depending on their activation state, this scar can serve to contain the injury and limit damage spread, but it may simultaneously act as a barrier to axonal regeneration.

Recent studies have demonstrated that astrocyte - derived exosomes (Astrocyte - Exos) play an important role in CNS injury and have become a growing focus in neuroregenerative research. These exosomes, enriched with various biologically active macromolecules, act as carriers that deliver functional cargo to both neighboring and distant recipient cells 205. This transfer can induce diverse functional changes, including neuroprotective effects through the regulation of neuronal uptake, differentiation, and activity 203, 206. Wu et al. reported that Astrocyte - Exos can target Toll - like receptor 7 (TLR7) to transport miR - 34c, thereby attenuating ischemia/reperfusion - induced brain injury by inhibiting the NF - κB/MAPK signaling axis 164. Long claimed that Astrocyte - Exos enriched with miR - 873a - 5p alleviated neurological deficits after TBI by suppressing ERK and NF - κB p65 phosphorylation and promoting the polarization of microglia toward the anti - inflammatory M2 phenotype 165. In both rat and mouse models, Zhang et al. showed that Astrocyte - Exos mitigated TBI - induced mitochondrial oxidative stress and neuronal apoptosis through activation of the Nrf2/HO - 1 signaling pathway 167. Despite these encouraging findings in TBI, to date, only one study has reported the application of Astrocyte - Exos in SCI, showing that they reduced fibrosis and improved functional outcomes 207. Given the shared injury mechanisms and therapeutic targets between TBI and SCI, it is likely that future research will increasingly explore the role of Astrocyte - Exos in SCI repair. Collectively, these findings suggest that Astrocyte - Exos hold significant promise as novel therapeutic agents for promoting functional recovery after SCI.

##### Schwann cells - derived exosomes

Schwann cells (SCs), a type of glial cell in the peripheral nervous system (PNS), play a critical role in the maintenance and regeneration of nerve fibers and have been extensively studied for their contributions to nerve repair 208, 209. Although they originate in the PNS, SCs are among the most widely investigated cell types due to their pivotal role in facilitating axonal regeneration and myelination. Notably, when transplanted into the spinal cord, SCs exhibit regenerative and myelination - supportive properties comparable to their activity in peripheral nerves 8, 210. Despite these advantages, the therapeutic efficacy of Schwann cell transplantation in promoting functional recovery after SCI remains limited. This is attributed to several key challenges, including the low survival rate of transplanted cells, the inability of SCs to traverse BBB, their poor migratory capacity within astrocytic tissue, and restricted axonal outgrowth beyond regions with high SC density 211. Proteomic analyses have identified several signaling pathways enriched and functionally relevant to the CNS microenvironment, including the neurotrophin, PI3K - Akt, and cAMP signaling pathways, all of which are involved in CNS repair processes 212. Furthermore, Schwann cell - derived exosomes (SCs - Exos) have been shown to promote axonal regeneration and attenuate inflammatory responses, offering promising therapeutic benefits for nerve tissue repair and inflammation reduction following neural injury.

Ren et al. demonstrated that SCs - Exos possess anti - inflammatory properties, suppressing M1 polarization while promoting M2 polarization of macrophages and microglia through the regulation of the SOCS3/STAT3 signaling pathway. These effects contribute to reduced neuronal apoptosis and improved motor function recovery following SCI 170. Pan et al. reported that SCs - Exos enhance functional recovery in SCI mice by reducing CSPG deposition and upregulating TLR2 expression on astrocytes via the NF - κB/PI3K signaling pathway 171. Furthermore, these exosomes inhibit PTP - σ activation by modulating the Rho/ROCK pathway, thereby limiting glial scar formation, promoting axonal regeneration, improving neuronal integrity, and facilitating locomotor recovery 168. Huang demonstrated that SCs - Exos could promote angiogenesis through the delivery of integrin - β1 213. Given that cell death represents a key feature of the post - injury microenvironment, SCs - Exos have also been shown to protect axons by enhancing autophagy and reducing apoptosis, potentially through the EGFR/Akt/mTOR signaling pathway 172. In addition, SCs - Exos help alleviate mitochondrial dysfunction and necroptosis via AMPK signaling pathway - driven mitophagy following SCI 169.

Overall, SCs - Exos have the comprehensive ability to restore the balance in the microenvironment after SCI. Thus, they hold significant potential as an innovative therapeutic approach for improving neurological functional recovery after neurotrauma.

##### Olfactory ensheathing cells - derived exosomes

Olfactory ensheathing cells (OECs), specialized glial cells within the olfactory system, play a vital role in supporting the regeneration of sensory neurons in the olfactory bulb. This is achieved through promoting axon growth and myelination. OECs are unique in their ability to facilitate nerve regeneration in both the central and peripheral nervous systems 15, 214. Due to these regenerative properties, OECs have been widely explored as a potential therapy for SCI, with demonstrated effects in promoting axonal regrowth and functional recovery. In recent years, OECs - derived exosomes (OECs - Exos) have garnered increasing interest as cell - free therapeutic agents for SCI. Tu et al. demonstrated that exosome - like vesicles derived from human OECs (hOECs - EV) promote NPC growth and alleviate t - BHP - induced oxidative toxicity. While the study was limited to *in vitro* experiments, the findings suggest that OECs - Exos enhance the proliferation and differentiation of NPCs, supporting their potential utility in regenerative applications 215. In a rat model of SCI, Fan et al. reported that OECs - Exos conferred neuroprotection by modulating the phenotype of macrophages and microglia via inhibition of the NF - κB and c - Jun signaling pathways, thereby reducing neuronal apoptosis 173. Additionally, OECs - Exos have been shown to deliver BDNF, which activates its receptor TrkB on neurons and counteracts TNF - α - induced apoptosis, further supporting neuronal survival and repair 174.

#### Non - neurologically derived exosomes

##### Mesenchymal stromal cell‑derived exosomes

Mesenchymal stromal cells (MSCs), which are multipotent stem cells derived from various tissues such as bone marrow, adipose tissue, and umbilical cord, have the capacity to differentiate into multiple cell types. This makes them valuable for tissue repair and regeneration 216-218. Moreover, MSCs possess immunomodulatory properties. These properties enable them to modulate immune responses, reduce inflammation, and support tissue healing. Due to these characteristics, MSCs have been widely investigated for their therapeutic potential in the treatment of SCI 6, 219. Emerging evidence indicates that the therapeutic benefits of MSCs primarily arise from their paracrine activity, rather than direct transdifferentiation or long - term engraftment. As such, MSC - derived exosomes (MSCs - Exos), which carry diverse paracrine mediators, have emerged as a promising acellular therapeutic strategy 220. Recent studies have highlighted the ability of MSCs - Exos to promote tissue regeneration, stimulate axonal growth, and inhibit glial scar formation, thereby enhancing neurological recovery following SCI 221.

MSCs - Exos are more readily available than those from many other cell types. Furthermore, their significantly smaller size compared to MSCs allows them to evade pulmonary and hepatic sequestration, enabling penetration of the BSCB. As a result, MSCs - Exos have gained considerable attention in the field of SCI therapy. Inflammation is a major pathological component of SCI, and reducing it is a key therapeutic strategy 222. Wang et al. reported that the miR - 17 - 92 cluster in bone marrow MSC (BMSCs) - Exos improves neurological function after SCI by mitigating inflammation and modulating neuronal apoptosis 223. Shao and colleagues showed that adipose - derived stem cell (ADSCs) - Exos more effectively reduce ROS levels and inflammatory cytokine expression following SCI 224. Luan et al. demonstrated that exosomes derived from umbilical cord - derived MSCs (UC - MSCs) protect against SCI by inhibiting inflammation through the NF - κB/MAPK signaling pathway, suggesting potential therapeutic targets 225. In addition, MSCs - Exos can modulate macrophage polarization to further suppress inflammation and support tissue repair. Chang et al. reported that microRNA - 125a in BMSCs - Exos promotes M2 macrophage polarization via IRF5 suppression 226. Moreover, inhibiting M1 microglia and promoting M2 microglia are also effective strategies for fostering functional recovery after SCI. ADSC - Exos inhibit M1 microglia, promote M2 microglial phenotypes, and activate the Nrf2/HO - 1 pathway, enhancing anti - inflammatory responses 227. Exosomes derived from human umbilical cord MSCs (hUC - MSCs) have also been shown to preserve BSCB integrity and improve functional recovery by upregulating junctional proteins and downregulating inflammatory mediators such as endothelin - 1 (ET - 1). Zhou et al. demonstrated that BMSCs reduce BSCB permeability, promote axonal regeneration, and accelerate motor function recovery by decreasing caspase - 1 expression and inhibiting IL - 1β release 228. Suppressing the activation of A1 neurotoxic reactive astrocytes can promote the function recovery after SCI. And the administration of BMSCs - Exos efficiently suppressed inflammation after traumatic SCI and suppressed the activation of A1 neurotoxic reactive astrocytes 229.

In summary, MSCs - derived exosomes contribute to SCI repair through multiple mechanisms, including promoting angiogenesis and axonal growth, regulating immune responses, inhibiting apoptosis, and preserving BSCB integrity. As a cell - free and immunologically safer alternative to MSCs transplantation, exosomes are unlikely to elicit strong immune reactions. Their therapeutic potential can be further enhanced through engineering approaches, such as the loading of specific microRNAs or therapeutic agents targeting SCI pathologies. However, further research is needed to clarify their long - term safety, stability, and off - target effects. Continued investigation into the detailed mechanisms and clinical translation of MSCs - Exos will be essential for advancing this promising field.

##### Pericytes - derived exosomes

Pericytes are specialized mural cells that reside within the walls of capillaries and microvessels, where they contribute to vascular stability, BBB integrity, and various repair mechanisms 230. Functionally, pericytes are essential for maintaining vascular homeostasis, supporting angiogenesis, and preserving BBB structure. They enhance endothelial cell function, promote vessel stabilization, and regulate cerebral blood flow through the secretion of numerous growth factors and regulatory molecules 231, 232. In the context of the CNS, pericytes play a particularly important role in maintaining BBB integrity and mitigating neuroinflammation, especially following traumatic injuries such as TBI and SCI 49, 233. Upon injury, pericytes respond by modulating inflammation and promoting tissue repair, making them a promising target for regenerative therapies in neurotrauma. In line with these roles, pericytes - derived exosomes (Pericytes - Exos) have also garnered increasing attention for their potential therapeutic applications.

Pericytes - Exos are produced and released under both physiological and pathological conditions, playing a critical role in mediating intercellular communication. Through the transfer of bioactive molecules, these exosomes enable pericytes to influence adjacent cells and regulate various biological processes, particularly those associated with injury response and tissue repair. In CNS injuries, Pericytes - Exos have been shown to reduce inflammation and oxidative stress, promote vascular regeneration, and facilitate neuronal recovery. Yuan et al. demonstrated that Pericytes - Exos exert therapeutic effects in SCI by mitigating pathological changes, improving motor function, and enhancing blood flow and oxygen delivery post - injury 234. These exosomes support endothelial regulation of circulation, preserve the integrity of the BSCB, and reduce edema. Mechanistically, they downregulate the expression of HIF - α, Bax, Aquaporin - 4, and MMP2, while upregulating Claudin - 5 and Bcl - 2, thereby inhibiting apoptosis. Under hypoxic conditions, Pericytes - Exos also protect spinal cord microvascular endothelial cells through activation of the PTEN/AKT signaling pathway. Additionally, Gao et al. reported that Pericytes - derived exosomes enriched with miR - 210 mitigate lipid peroxidation and preserve mitochondrial function, primarily by enhancing BBB integrity through the JAK1/STAT3 signaling pathway 235. By modulating this pathway, miR - 210 reduces inflammatory responses and reinforces structural components of the BBB, contributing to vascular stability and neural tissue repair. Collectively, these findings further elucidate the complex crosstalk between pericytes and endothelial cells in SCI and highlight Pericytes - Exos as a promising therapeutic target.

##### Macrophage‑derived exosomes

Macrophages are innate immune cells that play a pivotal role in maintaining tissue homeostasis, defending against pathogens, and facilitating wound repair. In CNS, macrophages are primarily present as microglia (resident macrophages) and infiltrating macrophages (recruited during injury or disease). These cells contribute to immune surveillance by detecting pathogens and cellular debris and are essential mediators of the inflammatory response. Following SCI, macrophages participate in clearing myelin debris, promoting angiogenesis, and supporting neural repair. However, their function is context - dependent and highly dynamic. Depending on environmental cues, macrophages may polarize into either pro - inflammatory (M1) or anti - inflammatory (M2) phenotypes. This plasticity underlies their dual roles in neuroinflammation and regeneration 59. Mounting researchers have suggested that M2 macrophages secrete anti - inflammatory cytokines and growth factors such as BDNF and vascular endothelial growth factor (VEGF). These mediators contribute to tissue repair by promoting axonal regeneration, stimulating angiogenesis, and modulating glial scar formation 236. M2 macrophages also support the resolution of inflammation and enhance remyelination 237. Furthermore, studies have shown that exosomes released by M2 macrophages during CNS injuries can promote vascular regeneration, cellular proliferation, and tissue repair 238.

Peng et al. reported that exosomes derived from M2 macrophages (Macrophages - Exos) increase the M2/M1 macrophage ratio via the miR - 23a - 3p/PTEN/PI3K/AKT signaling pathway. This modulation of the immune microenvironment facilitates SCI repair by promoting an anti - inflammatory state 239. Similarly, Zhang et al. demonstrated that peripheral Macrophages - Exos exert beneficial effects in SCI recovery by promoting microglial autophagy through suppression of the PI3K/AKT/mTOR pathway. This regulation enhances microglial polarization toward an anti - inflammatory phenotype, contributing to inflammation resolution 240. Huang et al. reported that M2 Macrophages - Exos enhance neurological recovery and angiogenesis following SCI, in part through activation of the HIF - 1/VEGF signaling pathway 241.

Moreover, studies have demonstrated that engineered Macrophages - Exos loaded with therapeutic agents can modulate the SCI microenvironment, offering promising treatment options. For SCI therapy, a novel nanomagnetic platform has been developed using click chemistry to functionalize the surface of M2 Macrophages - Exos with bioactive Ile - Lys - Val - Ala - Val (IKVAV) peptides. This engineered system reprograms macrophages toward an anti - inflammatory phenotype and promotes the neuronal differentiation of NSCs. In a murine SCI model, intravenous administration of these modified exosomes enabled targeted delivery to infiltrating macrophages at the injury site, leading to reduced pro - inflammatory cytokine expression and enhanced regeneration of neural tissue, ultimately improving motor function recovery 242. In another application, berberine - loaded M2 Macrophages - Exos have been employed as a targeted drug delivery system to transport berberine to the injured spinal cord. These exosomes facilitate the polarization of microglia from a pro - inflammatory M1 state to an anti - inflammatory M2 phenotype and suppress the production of inflammatory mediators. Additionally, they help inhibit neuronal apoptosis and support the restoration of motor function following SCI 243. Collectively, these studies highlight the potential of Macrophages - Exos as a promising strategy for targeted and effective SCI treatment.

##### Endothelial cell‑derived exosomes

Endothelial cells (ECs) in CNS are specialized components of BBB and BSCB, both of which are essential for maintaining CNS homeostasis. These cells are characterized by tight junctions, low transcytosis rates, and selective permeability, allowing them to protect neural tissue from toxins, pathogens, and immune cell infiltration while facilitating the exchange of nutrients and metabolic waste. Importantly, CNS ECs interact closely with neurons, astrocytes, pericytes, and immune cells to form the neurovascular unit (NVU), a critical structure that supports neural function and orchestrates the response to injury 244.

Following SCI, the integrity of the BSCB is compromised, leading to inflammation, oxidative stress, and neuronal damage. Studies have shown that spinal cord microvascular endothelial cells can promote axonal growth by downregulating miR - 323 - 5p expression. The reduction of this microRNA leads to the upregulation of genes associated with axon development, thereby facilitating axonal elongation and contributing to functional recovery after SCI 245. A recent study demonstrated that exosomes derived from vascular endothelial cells play an important role in promoting functional recovery after SCI. This effect is achieved by regulating communication between endothelial cells and microglia/macrophages through the delivery of USP13, a deubiquitinating enzyme with key roles in inflammation control. USP13 inhibits the ubiquitination and degradation of IκBα, a critical inhibitor of the NF - κB signaling pathway. By stabilizing IκBα, these exosomes suppress pro - inflammatory responses and promote the polarization of microglia and macrophages toward the anti - inflammatory M2 phenotype for SCI repair and neural regeneration 246. Despite these encouraging findings, limited research has been conducted on the application of endothelial cell - derived exosomes in SCI treatment, highlighting the need for further investigation to fully understand their therapeutic potential.

##### Induced pluripotent stem cell‑derived exosomes

Induced pluripotent stem cells (iPSCs) hold significant potential for treating SCI. Their capacity to differentiate into various neural cell types, such as neurons, astrocytes, and oligodendrocytes, enables the restoration of neural circuitry, promotion of axonal regeneration, and facilitation of remyelination in SCI models 279. Additionally, iPSCs can be derived from a patient's own somatic cells, thereby minimizing the risk of immune rejection. Nevertheless, several challenges hinder the clinical application of iPSC - based therapies. Due to their pluripotent nature, iPSC transplantation carries the risk of teratoma formation 280. Moreover, ensuring the precise differentiation and functional integration of iPSCs into damaged spinal cord tissue remains technically complex. Large - scale production of clinical - grade iPSCs and their derivatives also presents considerable logistical and regulatory challenges.

To address the limitations associated with iPSCs - based cell therapy, increasing attention has been directed toward the therapeutic use of iPSC - derived exosomes (iPSCs - Exos) in SCI. Azar Abbas and colleagues engineered exosomes derived from iPSCs that were enriched with miR - 23b, miR - 21 - 5p, and miR - 199b - 5p. These exosomes exhibited significant neuroprotective properties in SCI models by reducing inflammation and enhancing functional recovery. Notably, they exerted anti - inflammatory effects on neurons stimulated with LPS and IFN - γ, underscoring their potential as a cell - free therapeutic strategy for promoting spinal cord repair. 276. Moreover, iPSCs - Exos have been shown to enhance motor function in SCI mouse models. These exosomes promoted the polarization of macrophages from the pro - inflammatory M1 phenotype to the anti - inflammatory M2 phenotype, regulated inflammatory factor expression, and facilitated spinal cord recovery through the action of miR - 199b - 5p. This microRNA targetedHgf and modulated the PI3K signaling pathway to mediate therapeutic effects 275.

##### Regulatory T cell‑derived exosomes

Regulatory T cells (Tregs) play a crucial role in maintaining immune homeostasis by regulating inflammation and promoting tissue repair. Following SCI, Tregs have demonstrated therapeutic potential by modulating the inflammatory microenvironment, limiting secondary tissue damage, and supporting functional recovery 281. Nevertheless, the clinical application of Treg - based therapies in SCI faces several challenges. A major limitation lies in the difficulty of expanding and sustaining functional Tregs ex vivo while preserving their immunosuppressive activity. Additionally, systemic administration of Tregs may pose risks of broad immune suppression, potentially leading to increased susceptibility to infections or cancer if immune balance is disrupted. As a result, Treg - derived exosomes (Treg - Exos) have garnered increasing interest as a safer and more targeted alternative for SCI treatment.

Treg - Exos miR - 709 has been shown to attenuate microglial pyroptosis and promote motor function recovery following SCI 277. Moreover, Kong et al. demonstrated that exosomes secreted by Treg cells contribute to the repair of BSCB by reducing its permeability. This effect alleviates excitotoxic injury caused by inflammatory infiltration and ultimately supports motor function recovery. These exosomes exert their effects, in part, through enrichment of miR - 2861, which negatively regulates interleukin - 1 receptor - associated kinase 1 (IRAK1), a key mediator of the inflammatory response 282.

Collectively, these findings underscore the therapeutic potential of Treg - Exos in SCI. However, the underlying mechanisms by which these exosomes contribute to tissue repair and functional recovery remain incompletely understood, warranting further investigation.

### Mechanisms of exosomes - mediated treatment for SCI

As previously discussed, SCI results in a dysregulated microenvironment that exacerbates tissue damage and impedes neural regeneration. Due to their multifaceted roles in modulating the injury microenvironment, exosomes have demonstrated notable neuroprotective potential and have been extensively investigated in various preclinical SCI models for their ability to promote recovery. This section focuses on the underlying mechanisms through which exosomes contribute to SCI repair, with particular attention to their roles in regulating inflammation, promoting neuroprotection, and enhancing tissue regeneration. These findings underscore the therapeutic value of exosomes in restoring homeostasis and facilitating functional recovery after SCI (**Figure 6**).

#### Enhance the integrity of the BSCB

The BSCB is crucial for maintaining the microenvironment of the spinal cord. First, it restricts the entry of peripheral immune cells, thereby minimizing excessive inflammatory responses and protecting the spinal cord from immune - mediated injury. Second, as a neuroprotective barrier, the BSCB shields spinal neurons from potentially neurotoxic molecules present in the bloodstream. Third, it maintains ionic balance and molecular homeostasis, which are critical for proper neural signal transmission. After SCI, restoring the integrity of the BSCB is vital for minimizing secondary damage, optimizing the repair environment, and promoting tissue regeneration. Despite its protective role, however, the BSCB also poses a significant obstacle for therapeutic delivery. As such, innovative strategies, including nanotechnology and exosome - based delivery systems, are increasingly being explored to effectively transport therapeutic agents to the injured spinal cord. Maintaining BSCB integrity not only mitigates ongoing damage but also facilitates neural repair and enhances functional recovery following SCI. 283-285.

The BSCB primarily consists of endothelial cells, pericytes, astrocytes, and the basement membrane. Enhancing the function and interactions among these components can support BSCB integrity and contribute to SCI repair. BMSCs - Exos have been shown to preserve BSCB integrity and promote motor recovery after SCI by modulating the TIMP2/MMP signaling pathway. Specifically, tissue inhibitor of TIMP2 within these exosomes inhibits MMP activity, thereby increasing the expression of key tight junction proteins, including claudin - 5, occludin, zonula occludens - 1 (ZO - 1), and β - catenin, ultimately reducing BSCB damage 286. Similarly, exosomes from MSCs upregulate M2 macrophage markers, enhance TGF - β signaling, and increase tight junction protein expression, collectively reducing BSCB permeability 287. Additionally, maintaining the endothelial barrier in SCMECs under hypoxic conditions can aid in hindlimb functional recovery. NSCs - Exos loaded with FTY720, an immunomodulatory agent, preserve endothelial integrity via the PTEN/PI3K/AKT signaling pathway 153. Pericytes, as integral components of the neurovascular unit, are critical for maintaining BSCB structure. Enhancing pericytes function or increasing their number may improve SCI outcomes by strengthening the barrier. Treatment with BMSCs - derived exosomes has been shown to inhibit pericytes migration and reinforce BSCB integrity through the NF - κB/p65 signaling pathway 288. Moreover, exosomes can reduce edema and preserve BSCB function by suppressing pericytes pyroptosis via inhibition of the Nod1 inflammasome, thereby improving pericyte coverage and supporting recovery 155. Pericytes - Exos enriched with miR - 210 - 5p further stabilize the BSCB by attenuating lipid peroxidation and enhancing mitochondrial function through suppression of the JAK1/STAT3 signaling pathway 235.

#### Promote nerve regeneration

Neurons are the fundamental structural and functional units of the nervous system. Neural regeneration following SCI is a highly complex process involving neuronal outgrowth, axonal regeneration, and restoration of the local microenvironment.

In adults, the regenerative capacity of neurons following SCI is inherently limited. However, in preclinical models, various exosomes have demonstrated the ability to promote neuronal proliferation and regeneration. For instance, NSCs - Exos loaded with FTY720 enhance neuronal morphology through activation of the PTEN/AKT signaling pathway 153. Neurotrophic factor - enriched exosomes obtained from bone marrow BMSCs promote the differentiation of NSCs into neurons 155. Additionally, HpMSCs - Exos stimulate NSC proliferation via the MEK/ERK/CREB signaling cascade, increasing phosphorylation levels of MEK, ERK, and CREB 269. Axon extension represents a crucial step in neural regeneration. CD271^+^CD56^+^ BMSCs - Exos deliver MiR - 431 - 3p, which significantly enhances axonal elongation and branching in DRG neurons by targeting repulsive guidance molecule A (RGMa) 254. Furthermore, Microglia - Exos enriched with miR - 151 - 3p promote axonal regrowth and reduce neuronal apoptosis by inhibiting the p53/p21/CDK1 pathway 162. In summary, promoting axonal regeneration through exosome - mediated delivery of specific molecular signals represents a key strategy for SCI repair.

#### Promote angiogenesis

Neurogenesis and angiogenesis must work synergistically to accelerate regeneration following SCI. Restoring neurological function requires not only the regeneration of neural cells but also structural and vascular support from the extracellular matrix and surrounding blood vessels. Notably, the formation of new blood vessels is essential for tissue repair, as it ensures adequate oxygen and nutrient delivery to regenerating tissues. Exosomes play a critical role in promoting angiogenesis after SCI by stimulating endothelial cell proliferation and migration, as well as by regulating key angiogenesis - related signaling pathways.

After SCI, the preservation of endothelial cells helps limit secondary vascular damage, thereby ensuring the continued delivery of oxygen and nutrients necessary for restoring the microenvironment and neuronal circuitry. Vascular endothelial cells are particularly receptive to hypoxia - treated MSCs - Exos. Treatment with SCs - Exos has been shown to stimulate endothelial cell proliferation, migration, and tube formation. These effects collectively promote angiogenesis, reduce tissue damage, and enhance neurological recovery after SCI. Elevated HIF - 1α expression in hypoxia - stimulated MSCs - Exos enhances VEGF levels, suggesting a promising potential to enhance angiogenesis in SCI 289. Similarly, NSCs - Exos can significantly upregulate VEGF expression, thereby promoting SCMEC migration, proliferation, and tube formation processes in angiogenesis and tissue repair 156. M2 Macrophages - Exos also improve vascular regeneration and motor function recovery via activation of the HIF - 1α/VEGF signaling axis 241. Furthermore, miR - 126 - modified MSCs - Exos enhance microvascular regeneration and facilitate the migration of HUVECs by downregulating Sprouty - related EVH1 domain - containing protein 1 and phosphoinositide - 3 - kinase regulatory subunit 2 (PIK3R2) 290. In addition, macrophage membrane - fused exosome - mimetic nanovesicles have shown the potential to stimulate angiogenesis and selectively target ischemic endothelium, providing an innovative platform for targeted vascular repair following SCI 291.

Vascular regeneration is a complex process involving multiple signaling pathways that act synergistically to promote angiogenesis. These include the Wnt/β - catenin, PI3K/Akt, JNK/c - Jun, and PTEN/mTOR signaling cascades. Exosomes derived from M2 macrophages containing the ubiquitin isopeptidase OTULIN can activate the Wnt/β - catenin pathway by upregulating β - catenin expression. This stimulation, in turn, promotes the expression of angiogenesis - related genes in SCMECs 200. CSF - derived exosomes have also been shown to facilitate vascular regeneration and motor function recovery by activating the PI3K/Akt signaling pathway 292. Additionally, MSCs - Exos carrying siRNA targeting PTEN can suppress PTEN expression while promoting both axonal growth and neovascularization 293. Exosome - mimetic nanovesicles loaded with iron oxide nanoparticles (NV - IONPs) have been demonstrated to activate the JNK/c - Jun signaling axis, and their accumulation significantly contributes to the formation of new blood vessels following SCI 294.

#### Inhibit neuron apoptosis

Apoptosis, a form of programmed cell death, plays a critical role in the pathophysiology of SCI. It significantly impedes neurological recovery, primarily by inducing neuronal loss. Consequently, suppressing neuronal apoptosis is essential for enhancing therapeutic outcomes following SCI.

Various exosomes exhibit anti - apoptotic effects following SCI. BMSCs - Exos can protect against SCI by delivering miR - 181c, which reduces inflammation in microglia and spinal cord tissue via suppression of the PTEN/NF - κB signaling pathway. This results in decreased inflammation and neuronal apoptosis, ultimately improving recovery outcomes 295. Additionally, BMSCs - Exos promote motor function recovery by inhibiting neuronal apoptosis through the activation of the Wnt/β - catenin signaling pathway 296. NSCs - Exos attenuate SCI by initiating autophagic flux and suppressing apoptosis. miR - 374 - 5p, abundantly expressed in these exosomes, contributes significantly to neuroprotection by promoting autophagy and reducing apoptosis of injured neural cells 155. Furthermore, p53 has been identified as a target of miR - 151 - 3p in Microglia - Exos, implicating the p53/p21/CDK1 axis in the regulation of neuronal apoptosis and axonal regeneration 162. EPCs - Exos loaded with miR - 210 attenuate hypoxia/reoxygenation (H/R) - induced neuronal apoptosis and oxidative stress by modulating BDNF/TrkB and Nox2/Nox4 pathways 297. Overexpression of miR - 137 in EPCs - derived exosomes enhances neuroprotection by inhibiting apoptosis and mitochondrial dysfunction via the COX2/PGE2 signaling pathway 298. SCs - Exos containing MFG - E8 suppress neuronal apoptosis through activation of the SOCS3/STAT3 pathway 170.

Overall, exosomes have been shown to exhibit anti - apoptotic effects in various pathological conditions. These findings highlight the potential of exosome - based therapies as a promising strategy for reducing neuronal apoptosis and enhancing functional recovery following SCI.

#### Regulate inflammation

Excessive neuroinflammation impairs neuronal regeneration, thereby worsening the prognosis of patients with SCI. At the site of injury, persistent neuroinflammation disrupts axonal regeneration, hindering the re - establishment of neural connections and resulting in prolonged neurological deficits. Importantly, the inhibitory effects of inflammation and glial scarring following SCI are influenced, in part, by exosomes released from activated immune and glial cells.

According to previous studies, exosomes with anti - inflammatory effects can be classified based on their cellular origin or molecular content. During the initial stages of secondary damage in spinal cord tissue, exosomes contribute to neuroprotection by transporting bioactive molecules that reduce levels of ROS and inflammatory cytokines at the lesion site. For example, ADSCs - Exos carry LncGm37494, which significantly promotes SCI repair by downregulating inflammatory factor expression and improving functional outcomes 299. NSCs - Exos can deliver IGF - 1, which suppresses neuroinflammation by modulating the NF - κB signaling pathway 154. MiR - 544 - modified BMSCs - Exos can reduce the expression of pro - inflammatory cytokines such as IL - 1α, TNF - α, IL - 17β, and IL - 36β at the injury site following SCI 300. MicroRNAs delivered via exosomes can regulate inflammation through multiple signaling cascades. For instance, MSCs - Exos overexpressing miR - 145 - 5p inhibit the TLR4/NF - κB pathway, thereby attenuating the inflammatory response 268. Similarly, hypoxia - conditioned MSCs - Exos enriched with miR - 216a - 5p exert anti - inflammatory effects by targeting the TLR4/NF - κB/PI3K/AKT pathway and promoting microglial polarization 301.

Macrophage and microglial activity plays a pivotal role in regulating the inflammatory response following SCI. Various types of exosomes modulate this activity through multiple mechanisms, including inhibition of microglial and macrophage activation, suppression of M1 polarization, promotion of M2 polarization, and induction of autophagy in microglia. hUC - MSCs - Exos exhibit both anti - apoptotic and anti - inflammatory effects by suppressing the activation of microglia and macrophages through modulation of the BCL2/Bax and Wnt/β - catenin signaling pathways 302. Likewise, SCs - Exos containing milk fat globule - EGF factor 8 (MFG - E8) suppress M1 polarization and promote M2 polarization through the SOCS3/STAT3 pathway, thereby exerting anti - inflammatory effects 170. DPSCs - Exos have also been shown to reduce M1 macrophage polarization by modulating the ROS - MAPK - NFκB/p65 signaling pathway, contributing to inflammation resolution in SCI models 303. Furthermore, peripheral Macrophages - Exos enhance microglial autophagy by inhibiting the PI3K/AKT/mTOR pathway, thereby promoting microglial polarization toward an anti - inflammatory phenotype, which likely contributes significantly to the overall anti - inflammatory response 240.

Exosomes exert anti - inflammatory effects through multiple mechanisms. They can reduce ROS levels and suppress the production of inflammatory cytokines by delivering bioactive molecules to the injury site. Additionally, exosomes inhibit the activation of microglia and macrophages, suppress M1 polarization while promoting M2 polarization, and activate autophagy in microglia. Overall, exosomes and their cargo modulate several key molecular signaling pathways, contributing to the mitigation of the pathological microenvironment following SCI.

## Exosomes combined with biomaterials to repair SCI

**Figure 7:**
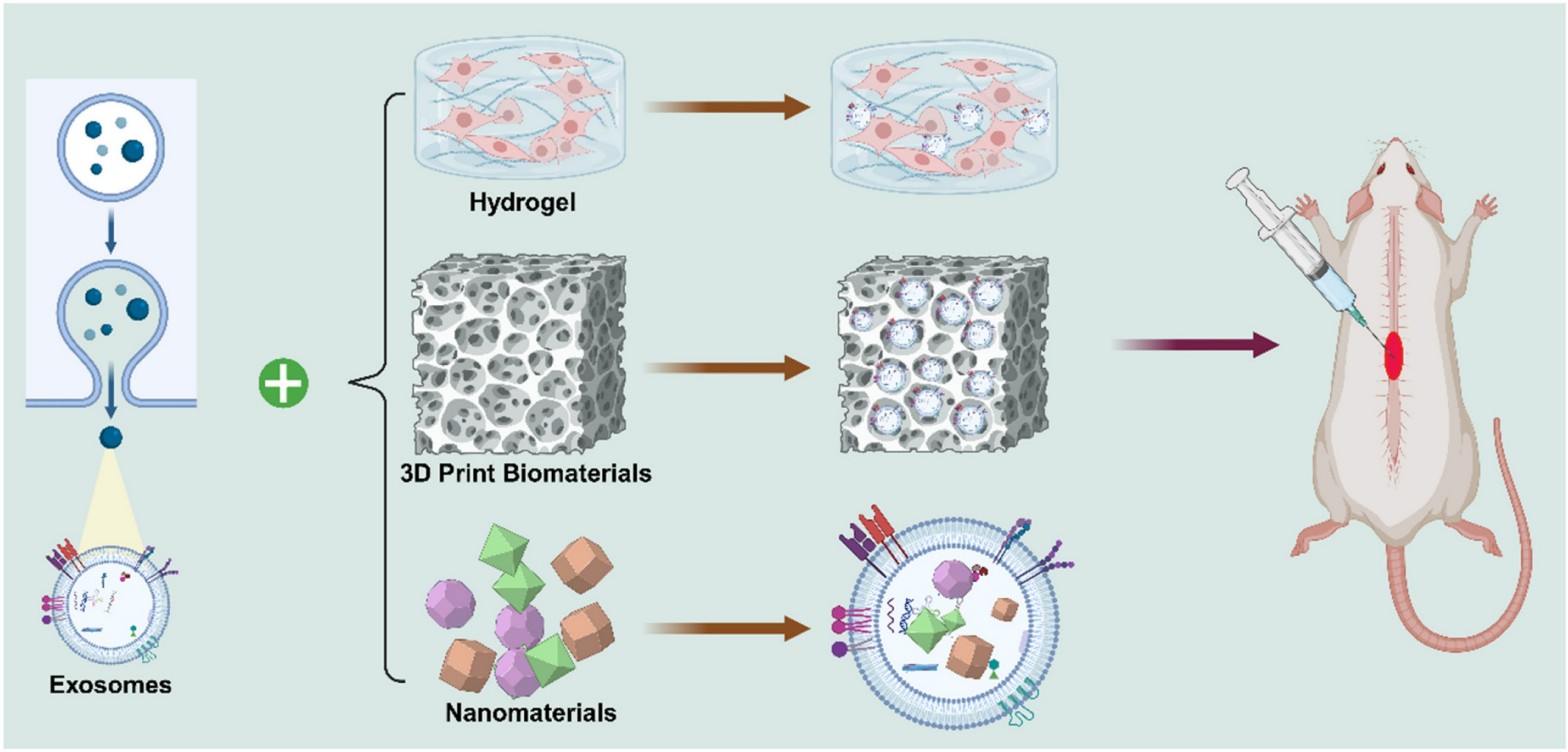
Schematic diagram of exosomes combined with biomaterials for the treatment of SCI. Exosomes can be combined with hydrogel, 3D print biomaterials, and nanomaterials respectively to treat spinal cord injury. Created with BioRender.com.

Exosomes have demonstrated promising therapeutic effects in the treatment of SCI. However, studies have identified several limitations associated with their intravenous administration, including low bioavailability, short circulation half - life, and poor targeting efficiency. Consequently, there is an urgent need to explore alternative strategies, particularly the integration of medical biomaterials, to enhance the therapeutic efficacy of exosome - based treatments. Biomaterials, primarily derived from natural or synthetic polymers, are widely utilized in tissue engineering and regenerative medicine. These materials can generally be categorized into three major types: hydrogels, 3D - printed scaffolds, and nanomaterials (**Figure 7**).

### Exosomes combined with hydrogels

Hydrogels are three - dimensional, hydrophilic polymers capable of retaining large amounts of water while closely mimicking the extracellular matrix (ECM). Their key properties, including biodegradability, adhesiveness, bioactivity, and tunable mechanical characteristics, make hydrogels ideal candidates for biomedical applications, particularly in tissue engineering and SCI repair 304. Acting as structural scaffolds, hydrogels support cellular growth, migration, and differentiation while providing a favorable environment for the sustained release of therapeutic agents.

In the context of SCI therapy, combining exosomes with hydrogels offers several advantages. Hydrogels serve as delivery vehicles, encapsulating exosomes and enabling controlled, localized release at the injury site, thus extending their therapeutic effect. Their high-water content supports cellular infiltration, which is an essential process for tissue regeneration. Moreover, hydrogels can be engineered to degrade gradually, allowing the time - dependent release of exosomes and other bioactive molecules necessary for neuronal repair.

Recent studies have explored exosome - loaded hydrogels in SCI models. For instance, electroconductive hydrogels loaded with M2 Microglia - Exos promote neural stem cell and axon growth in the dorsal root ganglion, regulate M2 microglial polarization, reduce acute inflammation, and enhance neuronal and axonal regeneration, significantly improving functional recovery in SCI rats 23. Similarly, BMSCs - Exos embedded in electroconductive hydrogels modulate M2 polarization via the NF - κB pathway, promote the neuronal and oligodendrocyte differentiation of NSCs, suppress astrocyte differentiation, and facilitate axon outgrowth through the PTEN/PI3K/AKT/mTOR pathway 305. Liu et al. developed a biomimetic magneto-electric hydrogel that integrates Fe_3_O_4_@BaTiO_3_ core-shell nanoparticles and HUMSC-Exos to enable remote, noninvasive electrical stimulation for the combined treatment of SCI. The Fe_3_O_4_@BaTiO_3_ nanoparticles are activated by an external magnetic field, generating electrical stimulation. This stimulation, coupled with the beneficial effects of HUMSC-Exos, effectively reduces early inflammatory responses following SCI and promotes the regeneration of new neurons and axons, thereby facilitating functional recovery after SCI 306. Additionally, injectable decellularized extracellular matrix hydrogels loaded with cortical Neuron - Exos have been shown to induce early M2 macrophage polarization, reduce neuronal apoptosis, and establish a pro - regenerative microenvironment in rodent SCI models 190.

Hydrogels also possess tunable physical properties, such as elasticity and stiffness, which can be optimized to match the mechanical characteristics of native spinal cord tissue, thereby enhancing tissue integration. Taken together, the incorporation of exosomes into hydrogel platforms represents a promising strategy to improve the efficacy of exosome - based therapies for SCI repair.

### Exosomes combined with 3D print

3D printing technologies have revolutionized tissue engineering by enabling the precise fabrication of scaffolds that replicate the complex architecture of biological tissues. The ability to create custom - designed scaffolds with tailored mechanical properties and geometries makes 3D printing a powerful tool for SCI repair 307. These scaffolds can be used to deliver exosomes in a spatially and temporally controlled manner, providing a supportive microenvironment for tissue regeneration.

The integration of exosomes with 3D printing represents a novel strategy for SCI therapy. Also known as additive manufacturing, 3D printing enables the construction of complex, biomimetic scaffolds that closely resemble the architecture and mechanical characteristics of native spinal cord tissue. Exosomes can be incorporated into these scaffolds and released gradually to facilitate cell migration, differentiation, and neuroprotection at the injury site. For instance, plant - derived exosomes loaded with isoliquiritigenin, when embedded within 3D - printed bionic scaffolds, have been shown to promote functional recovery and repair of spinal cord injury 308.

Furthermore, 3D printing allows for the creation of anatomically precise scaffolds, ensuring that the implanted construct fits the injury site accurately. This minimizes the risk of tissue rejection and enhances therapeutic efficacy. Overall, the combination of exosomes with 3D - printed scaffolds represents an exciting advancement in SCI repair, offering a promising platform for personalized regenerative medicine.

### Exosomes combined with nanomaterials

Nanomaterials are ideal for combination therapies with exosomes in SCI repair. This is because of their unique properties such as small size, high surface area, and the ability to interact with biological systems at the molecular level.

Nanomaterials can enhance the stability, bioavailability, and controlled release of exosomes, which in turn improves their effectiveness in promoting tissue regeneration and reducing inflammation. Recent studies have explored the application of exosome - loaded nanomaterials in SCI models. For instance, biocompatible macrophage - derived exosome - enclosed biomimetic manganese (Mn) - iron prussian blue analogues (MPBs) have been used for immunotherapy in SCI. These exosome - coated MPBs (E - MPBs) can enhance microglia accumulation, relieve the H_2_O_2_ - induced microenvironment, and inhibit apoptosis and inflammation *in vitro* 270. Moreover, a nanofiber scaffold combined with a hyaluronic acid hydrogel patch has been reported to release both exosomes and methylprednisolone. This dual - release system promotes macrophage polarization from the M1 to M2 phenotype, thus reducing inflammation, and inhibits apoptosis to enhance neuronal survival after SCI. The underlying mechanisms are related to the TLR4/NF - κB, MAPK, and Akt/mTOR signaling pathways 24.

In conclusion, the integration of nanomaterials with exosomes has great potential for enhancing the precision and efficacy of SCI therapies, which may lead to better patient outcomes.

## Engineering strategies for exosomes in SCI repair

Exosome engineering has emerged as a pivotal approach to enhance the therapeutic efficacy and specificity of exosome-based interventions for SCI. Various strategies have been developed to optimize the cargo content, targeting ability, and functional outcomes of exosomes.

Cargo loading techniques are commonly categorized into endogenous and exogenous methods. Endogenous loading involves modifying donor cells to naturally package desired therapeutic molecules into exosomes during biogenesis, such as through overexpression of specific RNAs or proteins 309. This method ensures stability and functional integration of the cargo. In contrast, exogenous loading directly introduces therapeutic agents into isolated exosomes using physical or chemical methods, including electroporation, sonication, extrusion, and chemical transfection 310, 311. These techniques allow for a wider range of cargos, such as siRNAs, miRNAs, mRNAs, proteins, or small-molecule drugs, to be efficiently incorporated into exosomes. Recent advancements have improved the efficiency and preservation of exosomal integrity during loading, making them promising carriers for neuroprotective and anti-inflammatory agents in SCI models 312.

Surface modification of exosomes has also been employed to enhance their targeting capability toward injured spinal cord tissue. Genetic engineering of donor cells to express targeting ligands, peptides, or antibodies on exosome membranes has shown promising results in improving lesion site accumulation. Alternatively, post-isolation chemical conjugation strategies, such as click chemistry and lipid insertion, have been utilized to functionalize exosome surfaces without significantly affecting their biophysical properties 258, 313.

Overall, the rational design and engineering of exosomes provide a promising avenue for overcoming the current limitations of natural exosome therapies, including poor targeting, low cargo loading capacity, and suboptimal therapeutic potency. Future work should focus on optimizing loading efficiency, maintaining exosome integrity, and ensuring scalable, reproducible manufacturing processes for clinical translation.

## Challenges in the application of exosomes to repair SCI

The application of exosomes in SCI repair faces several significant challenges. First, the isolation and purification of exosomes are complex processes. Yield, quality, and functional properties can vary depending on the cellular source and the isolation technique used. Therefore, standardizing exosome preparation protocols is essential to ensure consistency and reproducibility in therapeutic outcomes. Second, achieving targeted delivery to the injured spinal cord remains a major obstacle. Exosomes must traverse biological barriers and accumulate efficiently at the lesion site. Additionally, the potential immunogenicity and off - target effects of exosomes must be carefully assessed to avoid adverse immune responses or unintended tissue interactions.

Moreover, although exosomes exhibit generally low immunogenicity compared to cell therapies, recent studies suggest that their immunological safety should not be overlooked. Exosome membranes may retain immunogenic molecules from donor cells, and under pathological conditions or repeated administration, they could potentially elicit undesired immune responses. Therefore, careful characterization of exosomal surface markers and rigorous purification procedures are necessary to minimize immunogenicity risks. Long-term toxicity is another critical concern. Most current studies primarily assess short-term therapeutic effects, while data regarding the chronic biodistribution, clearance kinetics, and long-term safety of exosomes remain limited. Accumulation in non-target organs and potential off-target biological effects require thorough evaluation in future preclinical and clinical studies. Furthermore, exosome heterogeneity remains a major translational challenge. Exosomes derived even from the same cell type can exhibit substantial variability in size, cargo composition, and biological activity, depending on culture conditions, donor cell status, and isolation methods. Such heterogeneity may compromise the reproducibility and efficacy of exosome-based therapies. Therefore, standardized protocols for exosome production, isolation, and quality control are urgently needed to ensure consistent therapeutic outcomes.

Although exosomes have demonstrated neuroprotective and anti - inflammatory effects, their ability to fully regenerate damaged spinal cord tissue remains limited. Concerns regarding their stability, long - term therapeutic efficacy, and scalability for clinical - grade production continue to hinder widespread clinical translation. Addressing these challenges is critical to optimizing exosome - based therapies for SCI and realizing their full potential in regenerative medicine.

## Conclusions and future orientation

Exosome - based therapies hold great promise for modulating the pathological microenvironment following SCI. They have demonstrated the ability to promote the integrity of BSCB and angiogenesis, inhibit neuronal apoptosis, provide neuroprotection, facilitate axonal regeneration, exert anti - inflammatory effects, and support overall tissue repair. Nevertheless, the clinical translation of exosome - based approaches remains limited by challenges related to isolation, purification, targeting specificity, and delivery efficiency. Despite encouraging results in preclinical studies, the regenerative capacity and long - term efficacy of exosomes are still constrained. Moreover, although preclinical studies have demonstrated encouraging results, the clinical translation of exosome-based therapies for SCI remains in its early stages. A recent phase I clinical trial evaluated safety and potential effects of intrathecal injection of HUC - MSCs - Exos in complete subacute SCI, which demonstrated that intrathecal administration of allogeneic HUC - MSCs - Exos is safe in patients with subacute SCI. However, challenges such as scalable exosome production, standardization of isolation protocols, and regulatory compliance remain critical hurdles to overcome.

Moving forward, future research should prioritize the optimization of exosome isolation and purification methods to ensure reproducibility and high - quality production. Additionally, the development of efficient and precise delivery strategies is crucial to enhance exosome accumulation at the injury site. Innovations in exosome engineering, such as surface modification to improve cellular uptake and minimize immunogenicity, may further advance their clinical utility. Moreover, combining exosome - based therapies with gene editing technologies or stem cell interventions could synergistically enhance their regenerative potential.

To fully realize the therapeutic potential of exosomes in SCI and other neurodegenerative conditions, it is imperative to overcome existing technical barriers and deepen our understanding of exosome biology, biodistribution, and mechanisms of action.

## Figures and Tables

**Table 1:** The mechanism of neurologically cells - derived exosomes for regulating microenvironment after SCI.

Cell source	Highlighted Exosomes-associated cargo	Pathway	Mechanism of regulating microenvironment after SCI	Ref
Nerve Stem Cells	FTY720	PTEN/AKT pathway	Ameliorating the morphology of neurons	153
			Reducing inflammatory response	
			Inhibiting cell apoptosis	
Neural stem cell	miR-219a-2-3p	miR-219a-2-3p/YY1 pathway	Ameliorating inflammatory response	154
			Inhibiting cell apoptosis	
Neural stem cell	miR-374-5p	miR-374-5p/STK-4 axis	Inhibiting neural cell apoptosis	155
			Activating autophagy	
Neural stem cell	VEGF-A		Attenuating cell apoptosis	156
			Attenuating neuroinflammation	
			Activating of autophagy	
			Promoting angiogensis	
Neuron	miR-124-3p	PI3K/AKT/NF-κB pathway	Suppressing the activation of M1 microglia	157
			Suppressing neuroinfammation	
			Inhibiting A1 astrocytes.	
Neuron	miR-21-5p		Regulating microglia polarization	158
			Suppressing neuroinfammation	
M2 Microglia		NF-κB pathway	Enhancing neuronal survival and axon preservation	159
			Alleviating A1 astrocyte activation	
M2 Microglia	miR-672-5p	AIM2/ASC/Caspase-1 pathway	Promoting axon regeneration	160
			Reducing pyroptosis	
Microglia	miR‑615‑5p	miR‑615‑5p/MYRF	Promoting remyelination	161
Microglia	miR-151-3p	p53/p21/CDK1 pathway	Inhibiting neuronal apoptosis	162
			Promoting axonal regrowth	
Microglia		keap1/Nrf2/HO-1 signaling pathway	Modulating vascular regeneration	163
Astrocyte	miR-34c	NF-κB/MAPK	Inhibiting apoptosis	164
Astrocyte	miR-873a-5p	NF-κB pathway	Inhibiting microglial M1 phenotype	165
			Attenuating neuroinflammation	
Astrocyte	miR-148a-3p		Regulating the microglial phenotype	166
			Suppressing neuroinflammation	
Astrocyte		Nrf2/HO-1 pathway	Inhibiting neuronal apoptosis	167
Schwann cells		Rho/ROCK pathway	Reducing glial scar deposition	168
			Inducing neuronal axon growth	
Schwann cells		TLR4/NF-κB pathway MAPK pathway Akt/mTOR pathways	Increasing M2-type macrophages	24
			Reducing neuronal apoptosis	
Schwann cells		AMPK pathway	Reducing inflammatory response	169
			Suppressing necroptosis	
Schwann cells	MFG-E8	SOCS3/STAT3 pathway	Regulating macrophage/microglial polarization	170
Schwann cells		NF-κB/PI3K pathway	Inhibiting CSPGs deposition	171
Schwann cells		EGFR/Akt/mTOR pathway	Increasing autophagy	172
			Decreasing apoptosis	
Olfactory ensheathing cells		NF-κB and c-Jun signaling pathways	Switching the phenotype of macrophages/microglia	173
Olfactory ensheathing cells	BDNF		Preventing neuronal cells from apoptosis	174

**Table 2:** The mechanism of non-neurologically cells - derived exosomes for regulating microenvironment after SCI.

Cell source	Highlighted Exosomes-associated cargo	Pathway	Mechanism of regulating microenvironment after SCI	Reference
BMSCs	miR-219-5p	UBE2Z/NRF2 pathway	Inhibiting neuronal cells ferroptosis	247
BMSCs	miR‑17‑92		Alleviating apoptosis and inflammation	223
hUC-MSCs	miR-540-3p	SIX4/Yap1 pathway	Inhibiting the activation astrocytes Alleviating inflammation	248
ADSCs	circ-WDfy3	circ-WDfy3/miR-423-3p/GPX4 signaling pathway	Inhibiting ferroptosis and inflammatory response	224
BMSCs	Zinc finger and BTB domain-containing protein 4 (ZBTB4)	ZBTB4/ITIH3	Reducing neuronal apoptosis	249
			Repressing astrocyte activation	
BMSCs		IL-17 pathway	Inhibiting ferroptosis	250
UC-MSCs		NF-κB/MAPK pathways	Inhibiting inflammatory response	225
BMSCs	miR-26a-5p	BDNF-TrkB-CREB-KCC2 pathway	Inhibiting the inflammatory response and cell apoptosis	251
BMSCs	miR-21a-5p	miR-21a-5p/PELI1 axis	Inhibit macrophage/microglia pyroptosis	252
			Increasing antophagy	
BMSCs	Circ_0006640	circ_0006640/miR-382-5p/ IGF-1	Inhibiting microglial apoptosis	253
			Inhibiting inflammatory response	
CD271^+^ CD56^+^ BMSCs	miR-431-3p	miR-431-3p/RGMA axis	Promoting xon regeneration	254
ADSCs	LRRC75A-AS1	LRRC75A-AS1/FDFT1	Suppressing inflammatory response and apoptosis	255
hUCMSCs		ET-1	Maintaining BSCB's structural integrity	256
ADMSCs		Nrf2/HO-1 pathway	Ameliorating inflammatory response	227
			Regulating microglial polarization	
BMSCs	MiR-216a-5p	TLR4/NF-κB pathway	Suppressing inflammatory response	257
			Regulating microglia polarization	
RGD-CD146^+^CD271^+^ UCMSCs	miR-501-5p	miRNA-501-5p/ MLCK	Reducing BSCB destruction	258
BMSCs	miR-199a-5p	GSK-3β/β-catenin pathway	Promoting the proliferation of NSCs	259
BMSCs	MiR-137		Diminishing neuronal apoptosis	260
			Ameliorating inflammatory response	
HucMSCs		TLR2/MyD88/NF-κB/Rsad2	Restraining the activation of microglia	261
			Inhibiting inflammatory response	
BMSCs	MiR-146a		Diminishing neuronal apoptosis	262
			Ameliorating inflammatory response	
BMSCs	circZFHX3	mir-16-5p/IGF-1 axis	Repressing apoptosis and inflammatory response	263
MSCs	lncGm36569	Gm36569/ miR-5627-5p/FSP1	Suppressing neuronal cell ferroptosis	264
			Ameliorating inflammatory response	
BMSCs	miR-9-5p	HDAC5/FGF2 pathway	Alleviating apoptosis, inflammation and endoplasmic reticulum stress	265
ADSCs	miR-499a-5p	JNK3/c-jun-apoptotic signaling pathway	Alleviating apoptosis	266
BMSCs	miR-338-5p	Cnr1/Rap1/Akt pathway	Repressing cell apoptosis	267
			Promoting neuronal survival	
MSCs	miR-145-5p	TLR4/NF-κB pathway	Inhibiting inflammatory response	268
BMSCs	miR-26a	PTEN-AKT-mTOR pathway	Promoting axonal regeneration	269
			Improve neurogenesis	
			Attenuating glial scarring	
Pericyte		PTEN/PI3K/AKT pathway	Inhibiting cell apoptosis	234
			Promoting cell survival	
			Protecting blood-spinal cord barrier	
Pericyte	miR-210-5p	JAK1/STAT3 pathway	Improving BBB integrity	235
			Promoting angiogenesis	
M2 Macrophage			Enhancing anti-inflammatory response	270
			Promoting neuronal survival	
			Reducing the glial scar	
M2 Macrophage		HIF-1α/VEGF axis	Improving angiogenesis and neurogenesis	241
M2 Macrophage	OTULIN	Wnt/ β-catenin pathway	Promoting vascular regeneration	200
M1 Macrophages	miR-155	NF-κB/SOCS6/p65 pathway	Regulating the M1-polarized macrophages and microglia	271
Peripheral Macrophage		PI3K/AKT/mTOR pathway	Increasing autophagy	240
			Enhancing the polarization of anti-inflammatory type microglia	
M2 macrophage	miR-23a-3p	PTEN/PI3K/AKT pathway	Promoting M2 macrophage polarization	272
M2 macrophage	IKVAV peptides		Inhibiting inflammatory response	242
			Reducing infiltration of macrophages	
Microvascular endothelial cell	USP13	NF-κB pathway	Regulating microglia/macrophages polarization	246
Endothelial cell	miR199-5p	PI3K/AKT/PTEN pathway	Promoting nerve regeneration	273
			Maintaining repair-related phenotypes of Schwann cells	
iPSC-NSCs	let-7b-5p	let-7b-5p/LRIG3 pathway	Reducing inflammatory response	274
			Modulating microglial/macrophage pyroptosis	
iPSC	miR-199b-5p	miR-199b-5p/Hgf/PI3K pathway	Improving neural regeneration	275
			Regulating the polarization of macrophage	
iPSC	miR-23b, miR-21-5p, miR-199b-5p		Reducing inflammatory response	276
Regulatory T cell	miR-709	miR-709/ NKAP	Attenuating microglia pyroptosis	277
			Reducing inflammatory response	
Regulatory T cell	miR-2861	miR-2861/IRAK1	Promoting blood-spinal cord barrier repair	278
